# Acute and chronic effects of stretching on balance: a systematic review with multilevel meta-analysis

**DOI:** 10.3389/fmed.2024.1451180

**Published:** 2024-09-13

**Authors:** Lars Hubertus Lohmann, Astrid Zech, Gerit Plöschberger, Manuel Oraže, Daniel Jochum, Konstantin Warneke

**Affiliations:** ^1^Department of Human Movement Science and Exercise Physiology, University of Jena, Jena, Germany; ^2^Institute of Sport Science, Alpen-Adria University of Klagenfurt, Klagenfurt am Wörthersee, Austria; ^3^Viktor-Frankl Hochschule, Pädagogische Hochschule Kärnten, Klagenfurt am Wörthersee, Austria; ^4^Department of Health Sciences and Technology, ETH Zürich, Zürich, Switzerland; ^5^Institute of Human Movement Science, Sport and Health, University of Graz, Graz, Austria

**Keywords:** stretching, postural control, center of pressure, sway, Y-balance

## Abstract

**Introduction:**

Balance is a multifactorial construct with high relevance in, e.g., everyday life activities. Apart from sensorimotor control, muscle strength and size are positively linked with balance performance. While commonly trained for via resistance training, stretch training has emerged as a potential substitution in specific conditions. However, no review has investigated potential effects of stretching on balance, yet.

**Methods:**

PubMed, Web of Science and Scopus were searched with inception to February, 2024. Studies were included if they examined acute and/or chronic effects of any stretching type against passive and/or active controls on balance parameters – without any population-related restrictions concerning sex/gender, age, health status, activity level. Methodological quality was assessed using PEDro scale. Meta-analyses were performed if two or more studies reported on the same outcome. Certainty of evidence was determined based on GRADE criteria.

**Results:**

Eighteen acute and eleven chronic effect studies were included. Stretching studies exhibited significant improvements for sway parameters with eyes open against passive controls of moderate magnitude for chronic (ES: 0.63, *p* = 0.047) and of small magnitude for acute studies (ES: 0.21, *p* = 0.032). Most other subgroups against passive controls as well as actively-controlled comparisons resulted in trivial and/or non-significant effects.

**Conclusion:**

Even though some pooled effects slightly reached the level of significance, the overall results are biased by (very) low certainty of evidence (GRADE criteria downgrading for risk of bias, imprecision, publication bias). Moderators suggested by literature (strength, muscle size, flexibility, proprioception) were rarely assessed, which prevents conclusive final statements and calls for further, high quality evidence to clarify potential mechanisms–if any exist.

## 1 Introduction

The ability to restore and maintain postural control during upright standing or gait is of paramount importance to master everyday life activities in all age groups, however, especially in older adults and (orthopedic) patients (1). Considering balance a multifactorial construct, the literature indicates parameters such as sensorimotor control and muscle strength to be positively linked with balance performance (2, 3). While sensorimotor function was frequently triggered by performing a variety of exercises on unstable surfaces (4) or increasing complexity in the exercise execution, strength capacity is commonly enhanced via resistance training (5).

In order to maintain or prevent loss of motor function until older age there is growing interest to develop effective balance exercise routines. The demographic change toward an aging population poses several challenges on many societies with one in particular revolving around the enhanced need for medical care and nursing. Specifically, people above the age of 65 years (yrs.) are at particular risk for falls: One third of those as well as half of people >80 yrs. olds fall at least once a year (6). While falls are first and foremost a leading cause of fatal and non-fatal injuries (6, 7) that entail substantial medical costs (8), the fear of falling alone is associated with activity restrictions and may thus be an important contributor diminishing the quality of life of community-dwelling older adults (9). Several previous works were performed to determine moderators of balance, outlining, for instance, limited physical activity as a predictor of reduced balance (10). It is well known that sedentary behavior and reduced physical activity is closely related to impaired muscular performance and sarcopenia (11, 12), making the link between maximal strength and balance performance not surprising.

The relevance of reaching high force output in the lower extremity was underlined by Cattagni et al. (13) who described strength capacity as a discriminator between fallers and non-fallers. Accordingly, the American Geriatrics Society and American Academy of Orthopedic Surgeons panel on fall prevention listed muscular weakness and muscular function impairments as the most important risk factor for falls in older adults (14) and emphasized the demand for strength-increasing exercise routines since muscle weakness – including muscle size (15), strength (16) and flexibility (17) – moderates postural control. Consequently, the current 2022 “World guidelines for falls prevention and management for older adults” (18) strongly recommend the use of individualized progressive resistance training to reduce the risk of falls, while Chang et al. (19) recently reported further increases in balance ability when strength training is combined with acupuncture interventions.

However, to ensure a safe movement execution, meaning avoidance of adverse events while exercising, and to increase training effects especially in untrained and/or unexperienced populations, training supervision is strongly recommended (20). This requirement imposes severe burdens on conditioned and older participants, thereby restricting access to prevention but also rehabilitation programs, which is considered a major contributor to muscle function and size loss (21). Consequently, there seems to be high demand for balance exercise routines that forgo the need for travel and supervision, being time- and location-independent in its application, to improve exercise commitment and adherence rates.

Recent literature raised the possibility of interchangeable use of resistance training and high volume stretching under special circumstances: While full range of motion resistance training provided comparable flexibility increases (22), high volume and intensive stretching significantly increased strength and hypertrophy (23–25). Even though the required exercise durations to induce meaningful strength and muscle size adaptations seem time-consuming for stretching interventions, Behm et al. (26) proposed its usage as a relevant resistance training alternative when integrated into daily activities such as watching television or working in the office.

While recent literature discusses the potential of replacing strength training with high-volume stretching when aiming to increase muscle strength and size (27, 28), to date, no review has examined whether stretching has acute or chronic effects on balance. Thus, the objective of this systematic review with meta-analysis was to quantify the overall available evidence for the implementation of stretching to improve balance since the literature is too scarce for population-specific calculations – and thereby explore a possible new perspective for training and therapy.

## 2 Methods

The authors conducted the systematic review following the “Reporting Items for Systematic Reviews and Meta-Analyses 2020” (PRISMA 2020) guidelines (29) and opted to register the review in the PROSPERO database which, however, was rejected following the automatic processing with *possible* reasons given of which either one or more applied to this review: (a) review appears to be a scoping review, (b) review revolves around sporting performance, (c) review has insufficient information in fields (usually a lack of information on data extraction, risk of bias or data synthesis methods), (d) information was submitted in non-English language.

The search term was designed under consideration of the PICOS (Patient/Population, Intervention, Comparison, Outcomes, Study design) guidelines (30). The following eligibility criteria were applied to the literature search:

1. Randomized and non-randomized studies investigating any type of stretching meeting the definition criteria listed below in comparison to a passive (non-intervened) or active (all other types of exercise such as balance training, cycling, yoga or manual therapy) control condition.
2. Studies which investigated acute (immediately following an intervention) or chronic (long-term interventions of at least two weeks of stretching with a minimum of one session per week) stretching effects, including the most common stretching types [static, dynamic, ballistic and proprioceptive neuromuscular facilitation (PNF)] (31–33).
3. Works that quantified static and dynamic balance performance outcomes via, on the one hand Y-balance reach or the star excursion test, and on the other hand, center of pressure (COP) sway measurements.
4. Studies that recruited both healthy participants and/or patients, while no restrictions were applied regarding the target population concerning sex/gender, age, health status and activity/athletic status.

Studies were excluded for the following reasons:

- using combined interventions (stretching plus other exercise interventions except for warm-up through jogging or stationary bike),
- having uncontrolled study designs,
- lacking pre-post comparisons,
- investigating parameters on an ordinal scale [e.g., the Berg-Balance-Scale, see Lima et al. (34)],
- being of such low quality that vital aspects of the study design cannot be identified (e.g., lack of specificity regarding load control) and/or
- not being published in English-speaking, peer-reviewed journals.

The search strategy was developed based on the aforementioned eligibility criteria and applied to the three databases MEDLINE/PubMed, Web of Science, and Scopus (inception to February 2024) which was supplemented by manual search of the first 500 Google Scholar results as well as snowballing citation searching. The search terms were created based on individual database requirements, e.g., for PubMed:

(stretch*[Title/Abstract]) AND (balance[Title/Abstract] OR “postural control”[Title/Abstract] OR stability[Title/Abstract] OR proprioception[Title/Abstract]) NOT (pilates[Title/Abstract] OR dance[Title/Abstract] OR “stretch-shortening”[Title/Abstract])

The search strings for Web of Science and Scopus are listed in the Supplementary material. The databases were searched until 3 March 2024.

### 2.1 Stretching definitions for inclusion criteria

While there are several stretching definitions with broad extensions (22), the following definitions for static stretching, dynamic stretching and PNF were used to differentiate stretching from other interventions. In accordance with Behm (35) static stretching was defined as the lengthening of a muscle until stretch sensation/the point of discomfort and holding the muscle in a lengthened position, which can be performed passively by external devices, a partner or external weight, or actively by active movements. PNF is a stretching technique that incorporates a maximal voluntary contraction to a static stretching bout with or without antagonist contraction (contract-relax or contract-relax-antagonist-contract) (35). Interventions were considered dynamic stretching (36) if the exercise was performed as controlled back and forth movements in the end ROM, with ballistic stretching assumed a subcategory of dynamic stretching including less controlled, bouncing movements in the end ROM. Another specific subcategory of dynamic stretching was cyclic stretching, if participants performed constant velocity/angle stretching via a computerized system (37). All interventions that fit one of the aforementioned definitions were eligible for inclusion in this review.

### 2.2 Methodological study quality and risk of bias

PEDro rating was performed by two investigators (LHL & MO) in accordance with official guidelines (see Supplementary material) to assess the risk of bias (38). If no consensus was reached, a third author (KW) had the decisive vote. Additionally, risk of publication bias (39) was assessed by visual inspection of modified funnel plots, which was supplemented by the Egger’s regression test (40, 41).

### 2.3 Data processing and statistics

Study selection and data extraction were performed by GP, MO and DJ, and consequently double-checked by LHL and KW. Each record was screened by three reviewers independently. Data was extracted from the original studies into an Excel file (Microsoft 365, Microsoft Corp., Redmond, WA, USA) using a dual control principle via screen sharing. If a study did not contain means (M) and standard deviations (SD) in writing or in graphic illustrations, data were requested via e-mail or ResearchGate from the corresponding author of the respective study. If neither the corresponding author responded, nor there was another possibility to exactly determine M and SD, the study was excluded. Effect sizes (ES) were calculated based on MDiff from pre-to post-test by applying


$$M_{DIFF}=M_{\text{post}}-M_{\text{pre}}$$

while pooled SDs were determined by


$$SD_{\text{pooled}}=\sqrt{\frac{\left(n_{1}-1\right)*SD_{1}^{2}+\left(n_{2}-1\right)*SD_{2}^{2}}{\left(n_{1}-1\right)+\left(n_{2}-1\right)}}.$$

Accounting for multiple study (multiple outcomes of the same balance test, or different balance tests, or different stretching interventions tested against the same control) outcomes with co-variance originating from unknown sources, the robust variance estimation (RVE) meta-analysis calculation model (42) was used to pool the standardized mean differences (SMDs) and 95% confidence intervals (CIs) for acute and chronic stretching effects on balance. While comparisons with passive controls may indicate a general effectivity of the intervention, the attribution of potential effects to the specific intervention would require superior effects compared to other interventions (43). Thus, acute as well as chronic stretching effects were also compared to active, alternative control conditions. Due to the highly specific nature of balance tasks and subsequently differences that each task places on the underlying abilities needed, separate analyses were performed based on the test used to obtain the balance outcomes. Therefore, we differentiated 3 subgroups as (1) combining Y-Balance test (YBT), Star Excursion (SEBT) (and Forward reach test (FRT)), (2) sway/COP eyes open, and (3) sway/COP eyes closed. The rationale for dividing the analyses into static (sway/COP open eyes, sway/COP closed eyes) and dynamic (YBT/SEBT/FRT) balance lies in the task specificity of tests and different underlying control mechanisms such as the involvement of the neuromuscular system (44, 45). While YBT/SEBT(/FRT) demand control of the body’s center of gravity during dynamic, one-legged movement/reach, sway/COP tests quantify the extent of sway during static standing. Hereby, open-eyes and closed-eyes conditions were distinguished due to the known impact of visual feedback on static standing balance (46) that increases reliance on vestibular and proprioceptive input during eyes-closed balance performance (47). Improving postural control is of particular interest in people with functional limitations such as in older adults and patients with chronic diseases.

If possible, the analyses were further refined for stretching types (static, dynamic, PNF). To further reduce intra-study heterogeneity, stability index outcomes were removed for sensitivity analyses.

Outcome heterogeneity was assessed with τ^2^ and categorized in accordance to pooled effect sizes (ES), interpreted as follows: trivial: 0 ≤ ES < 0.2; small: 0.2 ≤ ES < 0.5; moderate: 0.5 ≤ ES < 0.8; and large: ES ≥ 0.8 (48). All calculations were performed using R (version 4.2.3) with the robumeta and meta package (42) under special consideration of the study design (parallel and cross-over design).

### 2.4 Certainty of evidence

The certainty of evidence was rated adhering to the GRADE working group criteria with categorizations as “very low” (effect estimate very uncertain), “low” (further research is very likely to change the effect estimate), “moderate” (further research is likely to change the effect estimate), or “high” (further research is very unlikely to change the effect estimate) (49). Accordingly, certainty is downgraded for risk of bias (limitations in study design and execution, e.g., lacking allocation concealment or blinding of subjects and/or investigators), inconsistency of results (unexplained heterogeneity of results assessed, e.g., via τ^2^), indirectness of evidence (evidence stems from research that does not directly compare the interventions of interest, is not delivered to the populations of interest, and/or does not measure the outcomes of interest), imprecise data (generally, if studies include only few participants and events and have a wide CI around the estimate of the effect, e.g., when the 95%CI overlaps no effect and CI fails to exclude important harm or benefit), and publication bias (systematic over- or underestimation due to selective publication of studies assessed, e.g., via Egger’s regression and/or funnel plots), while strong evidence of association (large or very large magnitude of effect with good precision regarding the CI – see above), evidence of a dose–response gradient, and plausible confounders (confounding is expected to have influenced the result in a way that the effect is even higher when adjusted for the confounders, e.g., when confounding is expected to have reduced a demonstrated (large magnitude) effect) enabled an upgrade.

## 3 Results

**Figure 1:**
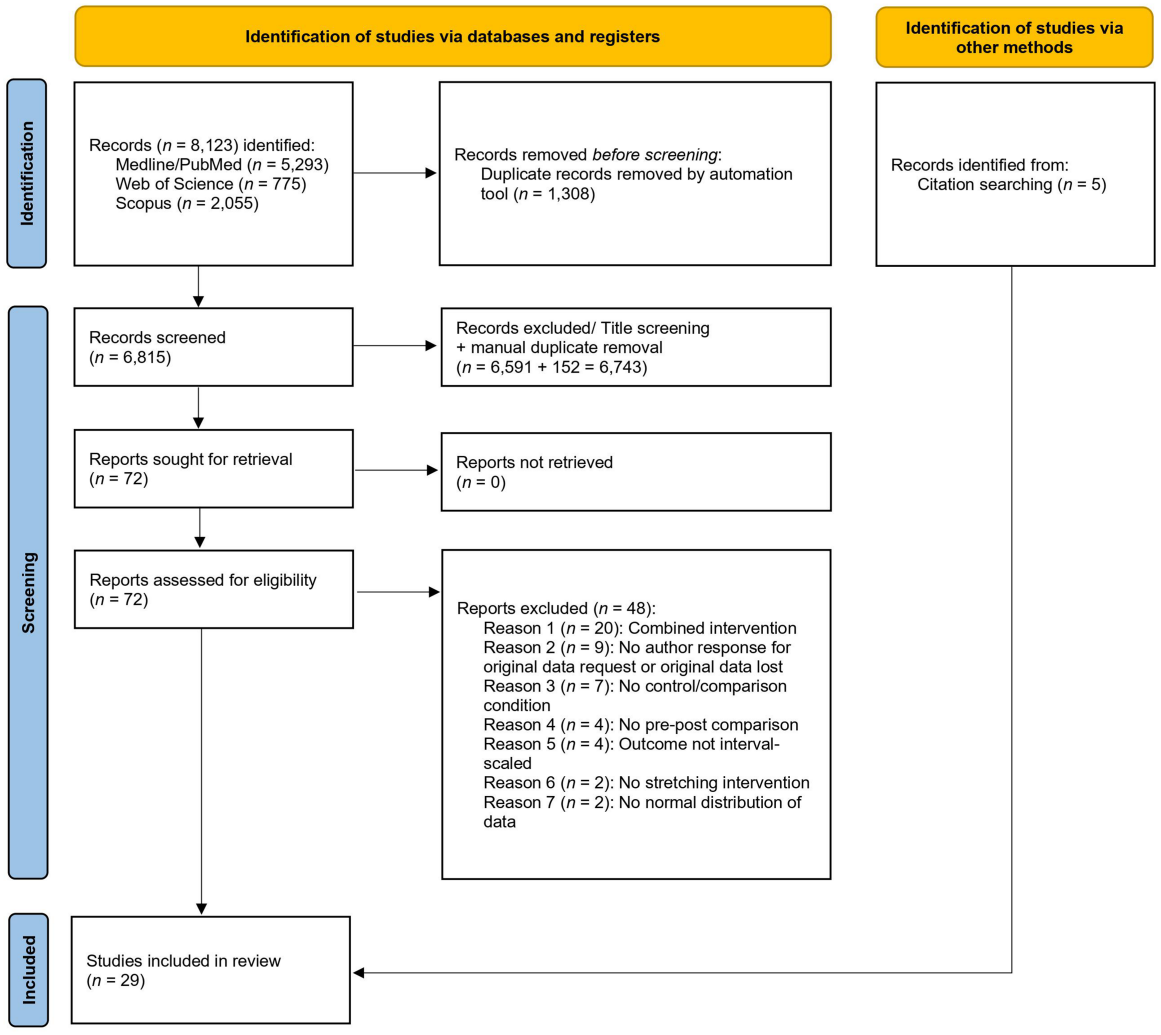
PRISMA flow-chart diagram.

**Figure 2:**
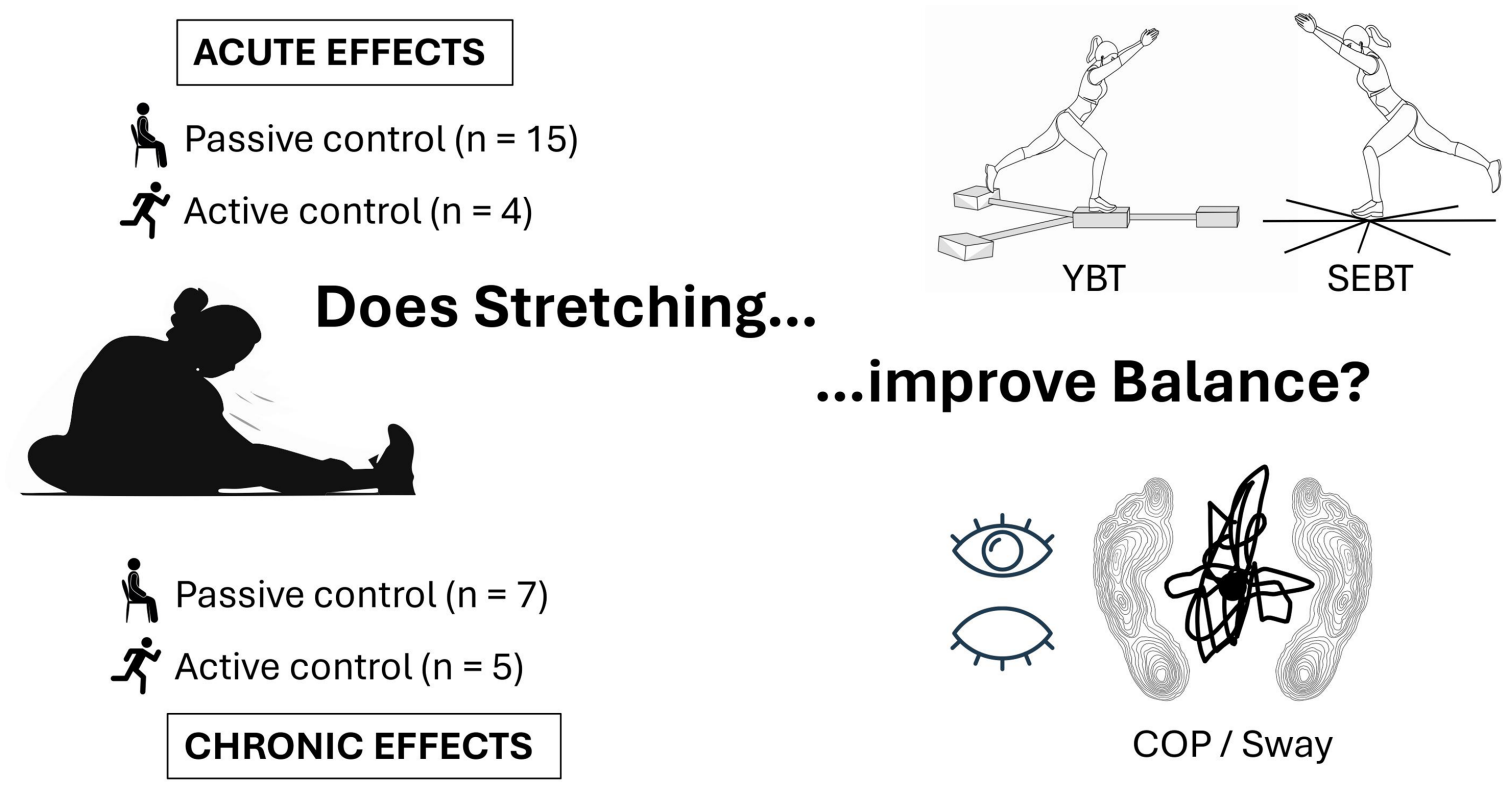
Overview of included studies.

The flow chart (Figure 1) illustrates the literature search, that resulted in a total of 29 (50–78) studies of which 18 (50, 52–59, 61, 63, 65, 67, 68, 71, 73–75) investigated acute and eleven (51, 60, 62, 64, 66, 69, 70, 72, 76–78) chronic effects. Fifteen acute effect studies compared stretching to a passive control while four opposed stretching to an active control. For chronic effects, seven studies compared stretching groups to passive and five studies to active control groups (see Figure 2). More detailed study descriptions can be found in Table 1.

**Table 1:** Study characteristics.

Study	Tags*	Participants	Intervention	Measurement equipment	Outcome (M ± SD)
Akdag et al. (50)	Acute Active Adults Healthy	*n* = 36 (no info on sex), age: 23.45 ± 2.49 yrs., height: 172.2 ± 0.01 cm, weight: 70.9 ± 15.5 kg. Parallel group design with random allocation (2 groups each *n* = 18). No information on training level. Healthy individuals with hip flexor tightness.	Interventions: Dynamic stretching and self-mobilization (active CG). Muscle(s): Hip flexors. Dynamic protocol: Unilateral stretch 6 sets 10×2 s. Self-mobilization protocol: Unilateral mobilization 6 sets 10×2 s.	Y-balance test (tape on floor)	*YBT anterior reach (in % of leg length) right leg:* DS Pre: 110.9 ± 16.2, Post: 122.1 ± 22.5 SM Pre: 102.4 ± 13.914, Post: 115.9 ± 21.4 YBT anterior reach (in % of leg length) left leg: DS Pre: 115.5 ± 25.1, Post: 126.1 ± 24.9 SM Pre: 112.7 ± 19.1, Post: 120.9 ± 19.3 *YBT posteromedial reach (in % of leg length) right leg:* DS Pre: 119.3 ± 25.2, Post: 128.6 ± 26.8 SM Pre: 122.4 ± 18.0, Post: 132.6 ± 19.0 *YBT posteromedial reach (in % of leg length) left leg:* DS Pre: 123.1 ± 24.5, Post: 132.0 ± 27.0 SM Pre: 122.1 ± 19.5, Post: 132.3 ± 18.6 *YBT posterolateral reach (in % of leg length) right leg:* DS Pre: 106.8 ± 15.6, Post: 118.7 ± 21.3 SM Pre: 106.1 ± 15.4, Post: 118.1 ± 22.7 *YBT posterolateral reach (in % of leg length) left leg:* DS Pre: 115.5 ± 22.1, Post: 123.9 ± 25.7 SM Pre: 105.5 ± 21.2, Post: 122.1 ± 24.7 Significant increase in YBT performance in both groups with no significant difference between them.
Alahmari et al. (51)	Chronic Passive Adults Patients	*n* = 60 (M = 60), age: 25.7 ± 5.9 yrs., height: 166 cm, weight: 70.75 ± 14.5 kg. Parallel group design with random allocation (3 groups each *n* = 20). No information on training level. All participants with ankle sprain within the 3 months prior to the intervention.	Interventions: PNF stretching, TENS-PNS stretching and non-intervened CG. Muscle(s): Triceps surae. PNF protocol: 4×50 s (20 s contraction, 30 s stretch) on the affected lower limb. TENS-PNF protocol: Same as PNF plus TENS during contraction (50 Hz, 250 microsecond pulse duration). CG protocol: No intervention. Intervention period: 4 sessions/week for 4 weeks.	Star Excursion Balance Test (tape on floor)	*SEBT anterior reach (in % of leg length)* TENS-PNF Pre: 78.2 ± 2.5, Post 3-weeks: 81.9 ± 3.1, Post 5-weeks: 82.5 ± 2.6 PNF Pre: 68.5 ± 3.9, Post 3-weeks: 70.2 ± 4.1, Post 5-weeks: 69.4 ± 4.2 CG Pre: 70.5 ± 5.9, Post 3-weeks: 70.7 ± 7.0, Post 5-weeks: 70.8 ± 7.3 *SEBT posterior reach (in % of leg length)* TENS-PNF Pre: 92.4 ± 3.1, Post 3-weeks: 96.1 ± 3.1, Post 5-weeks: 96.3 ± 3.1 PNF Pre: 91.2 ± 4.6, Post 3-weeks: 92.4 ± 4.5, Post 5-weeks: 92.3 ± 4.7 CG Pre: 90.7 ± 5.4, Post 3-weeks: 90.9 ± 5.3, Post 5-weeks: 91.0 ± 5.2 *SEBT medial reach (in % of leg length)* TENS-PNF Pre: 95.7 ± 3.5, Post 3-weeks: 99.8 ± 4.1, Post 5-weeks: 100.1 ± 4.1 PNF Pre: 96.9 ± 3.7, Post 3-weeks: 98.3 ± 3.5, Post 5-weeks: 98.1 ± 3.7 CG Pre: 96.6 ± 3.5, Post 3-weeks: 96.8 ± 3.3, Post 5-weeks: 96.7 ± 3.4 *SEBT lateral reach (in % of leg length)* TENS-PNF Pre: 89.1 ± 5.9, Post 3-weeks: 92.1 ± 5.9, Post 5-weeks: 92.2 ± 5.7 PNF Pre: 92.9 ± 4.7, Post 3-weeks: 93.9 ± 4.2, Post 5-weeks: 93.4 ± 4.1 CG Pre: 91.6 ± 5.5, Post 3-weeks: 91.8 ± 5.4, Post 5-weeks: 91.8 ± 5.1 *SEBT anterolateral reach (in % of leg length)* TENS-PNF Pre: 74.9 ± 4.7, Post 3-weeks: 77 ± 4.6, Post 5-weeks: 77.3 ± 4.7
					PNF Pre: 75.1 ± 3.9, Post 3-weeks: 76.4 ± 3.8, Post 5-weeks: 75.6 ± 4.0 CG Pre: 75.8 ± 4.1, Post 3-weeks: 76.1 ± 4, Post 5-weeks: 76.1 ± 4 *SEBT anteromedial reach (in % of leg length)* TENS-PNF Pre: 84.2 ± 5.8, Post 3-weeks: 88.1 ± 5.9, Post 5-weeks: 88.7 ± 5.8 PNF Pre: 83.1 ± 5.7, Post 3-weeks: 84.3 ± 5.7, Post 5-weeks: 84.2 ± 5.4 CG Pre: 83.8 ± 5.7, Post 3-weeks: 84.1 ± 5.8, Post 5-weeks: 84.1 ± 5.8 *SEBT posterolateral reach (in % of leg length)* TENS-PNF Pre: 95.1 ± 3, Post 3-weeks: 98.6 ± 2.4, Post 5-weeks: 98.9 ± 2.4 PNF Pre: 95.6 ± 2.9, Post 3-weeks: 96.7 ± 3.2, Post 5-weeks: 96.3 ± 3.4 CG Pre: 94.9 ± 2.8, Post 3-weeks: 95.1 ± 2.9, Post 5-weeks: 95.1 ± 2.9 *SEBT posteromedial reach (in % of leg length)* TENS-PNF Pre: 96.9 ± 2.6, Post 3-weeks: 102.1 ± 4, Post 5-weeks: 102.3 ± 4 PNF Pre: 96.8 ± 2.8, Post 3-weeks: 98.3 ± 3.2, Post 5-weeks: 97.7 ± 3.3 CG Pre: 96.9 ± 3.3, Post 3-weeks: 97.2 ± 3, Post 5-weeks: 97.1 ± 3 Significant SEBT increase for TENS-PNF compared to PNF and CG.
Alimoradi et al. (62)	Chronic Passive Adults Healthy	*n* = 45 (*F* = 45), age: 22.98 ± 1.45 yrs., height: 169.6 ± 5.3 cm, weight: 53.62 ± 2.69 kg. Parallel group design with random allocation (3 groups each *n* = 15). Youth athletes from provincial soccer teams.	Interventions: Dynamic stretching and non-intervened CG. Muscle(s): Hamstrings, quadriceps, gastrocnemius (& soleus). IG1 protocol: Bilateral stretching of hamstring, quadriceps & gastrocnemius muscle 3×30 s. IG2 protocol: Same as IG1 plus soleus stretching 3×30 s. CG protocol: No intervention. Intervention period: 12 sessions within 4 weeks.	Y-balance test via OctoBalance device (Check your Motion, Albacete, Spain)	*YBT reach (in % of leg length)* IG1 Pre: 78.6 ± 3.3, Post: 84.38 ± 2.9 IG2 Pre: 78.65 ± 2.5, Post: 86.35 ± 2.2 CG Pre: 77.83 ± 2.3, Post: 78.2 ± 2.1 Significant improvement for both IGs compared to CG in Y-balance test. No difference between IGs.
Ayán et al. (72)	Chronic Active Elderly Patients	*n* = 23 (*F* = 7, M = 16), age: 68.04 ± 7.86 yrs., height: not reported, weight: not reported.	Interventions: Stretching and Hatha yoga (active CG).	Sway area in standing with eyes open via Stabilometer (no info about device).	*Sway area (mm^2^):* Stretching Pre: 118.8 ± 67.9, Post: 104.1 ± 38.7 Yoga Pre: 101.4 ± 49.6, Post: 93.9 ± 42.1 *Path length (mm):* Stretching Pre: 254.0 ± 103.3, Post: 261.3 ± 118.3
		Parallel group design with random allocation (2 groups, stretching *n* = 11, Yoga *n* = 12). Patients with Parkinson’s disease	Muscle(s): Sternocleidomastoid, splenius, trapeze, triceps, posterior shoulder capsule, paravertebral, hamstrings, quadriceps, gastrocnemius, soleus and tibialis anterior Stretching protocol: 10 min warm-up, 40 min of stretching, 10 min diaphragmatic breathing. No information on number of stretching exercises. Each exercise for 2×15 s. Hatha yoga protocol: 10 min warm-up, 30 min yoga (standing, sitting, and supine positions), 20 min breathing. Intervention period: 1 session/week for 8 weeks.		Yoga Pre: 214.4 ± 87.4, Post: 217.3 ± 83.6 No significant difference from pre to post in both groups. No significant difference between groups.
Behm et al. (73)	Acute Passive Adults Healthy	*n* = 16 (M = 16), age: 24.1 ± 7.4 yrs., height: 172.3 ± 6.5 cm, weight: 71.5 ± 15.4 kg. Cross-over study with random sequence allocation (2 groups). No info on training level. University students.	Interventions: Static stretching and non-intervened CG. Muscle(s): Quadriceps, hamstrings, gastrocnemius, soleus. Static protocol: Unilateral stretch of the 4 muscles 3×45 s. CG protocol: No intervention.	Contacts via Wobble board (Kinematic Measurement Systems, Muncie, IN, USA).	*Number of wobble board contacts* SS Pre: 8.8 ± 1.7, Post: 9 ± 1.8 CG Pre: 10.8 ± 2, Post: 8.9 ± 1.5 Significant decrease in performance for SS compared to CG.
Coratella et al. (74)	Acute Passive Adults Healthy	*n* = 38 (*F* = 19, M = 19), age: 26 ± 3 yrs., height: 173 ± 10 cm, weight: 69 ± 17 kg. Cross-over study with random sequence allocation (2 groups). Recreationally active.	Static stretching and non-intervened CG. Muscle(s): Hip flexors, hip extensors, plantar flexors, plantar extensors. Static protocol: Unilateral, alternating stretch with 4 exercises 5×45 s. CG protocol: No intervention.	Bipedal balance via computerized stabilometry platform (Prokin 252, Tecnobody, Bergamo, Italia). Balance foam pad (model LivePro 48x40x6cm, Nanotong Liveup Sports Co. Ltd.,	*Static test COP sway area (mm^2^) eyes open:* Static stretch Pre: 349 ± 75, Post-immediate: 353 ± 80, Post-15 min: 350 ± 77, Post-30 min: 351 ± 76 CG Pre: 352 ± 77, Post: 349 ± 79, Post-15 min: 349 ± 74, Post-30 min: 350 ± 75 *Static test COP sway area (mm^2^) eyes closed:* Static stretch Pre: 463 ± 106, Post-immediate: 470 ± 110, Post-15 min: 467 ± 107, Post-30 min: 467 ± 106 CG Pre: 467 ± 103, Post-immediate: 468 ± 109, Post-15 min: 468 ± 107, Post-30 min: 468 ± 111 *Static test COP sway area (mm^2^) eyes open + foam pad:* Static stretch Pre: 436 ± 89, Post-immediate: 441 ± 91, Post-15 min: 438 ± 89, Post-30 min: 443 ± 94
				Nantong, China). Static and dynamic conditions.	CG Pre: 439 ± 91, Post-immediate: 434 ± 89, Post-15 min: 432 ± 87, Post-30 min: 441 ± 99 *Static test COP sway area (mm^2^) eyes closed + foam pad:* Static stretch Pre: 596 ± 111, Post-immediate: 596 ± 110, Post-15 min: 598 ± 110, Post-30 min: 599 ± 112 CG Pre: 598 ± 109, Post-immediate: 600 ± 122, Post-15 min: 590 ± 109, Post-30 min: 600 ± 122 *Dynamic test COP sway area (mm^2^) eyes open:* Static stretch Pre: 1164 ± 248, Post-immediate: 1165 ± 223, Post-15 min: 1143 ± 233, Post-30 min: 1153 ± 248 CG Pre: 1156 ± 251, Post-immediate: 1151 ± 262, Post-15 min: 1151 ± 244, Post-30 min: 1154 ± 247 *Dynamic test COP sway area (mm^2^) eyes closed:* Static stretch Pre: 1533 ± 352, Post-immediate: 1548 ± 370, Post-15 min: 1532 ± 359, Post-30 min: 1138 ± 351 CG Pre: 1541 ± 340, Post-immediate: 1543 ± 359, Post-15 min: 1546 ± 353, Post-30 min: 1546 ± 366 *Dynamic test COP sway area (mm^2^) eyes open + foam:* Static stretch Pre: 1440 ± 294, Post-immediate: 1461 ± 289, Post-15 min: 1422 ± 281, Post-30 min: 1446 ± 301 CG Pre: 1448 ± 301, Post-immediate: 1434 ± 294, Post-15 min: 1425 ± 287, Post-30 min: 1455 ± 325 *Dynamic test COP sway area (mm^2^) eyes closed + foam pad:* Static stretch Pre: 1960 ± 368, Post-immediate: 1964 ± 412, Post-15 min: 1976 ± 394, Post-30 min: 1971 ± 384 CG Pre: 1974 ± 361, Post-immediate: 1981 ± 402, Post-15 min: 1947 ± 360, Post-30 min: 1979 ± 401 *Static test COP sway perimeter (mm) eyes open:* Static stretch Pre: 385 ± 77, Post-immediate: 360 ± 86, Post-15 min: 361 ± 80, Post-30 min: 365 ± 81 CG Pre: 387 ± 85, Post-immediate: 384 ± 82, Post-15 min: 366 ± 78, Post-30 min: 375 ± 77 *Static test COP sway perimeter (mm) eyes closed:* Static stretch Pre: 497 ± 111, Post-immediate: 498 ± 119, Post-15 min: 495 ± 111, Post-30 min: 486 ± 114 CG Pre: 486 ± 113, Post-immediate: 491 ± 114, Post-15 min: 491 ± 113, Post-30 min: 482 ± 114 *Static test COP sway perimeter (mm) eyes open + foam pad:* Static stretch Pre: 473 ± 92, Post-immediate: 463 ± 97, Post-15 min: 464 ± 93, Post-30 min: 474 ± 100 CG Pre: 461 ± 96, Post-immediate: 460 ± 93, Post-15 min: 459 ± 91, Post-30 min: 472 ± 103 *Static test COP sway perimeter (mm) eyes closed + foam pad:* Static stretch Pre: 605 ± 113, Post-immediate: 608 ± 117, Post-15 min: 622 ± 113, Post-30 min: 617 ± 120 CG Pre: 616 ± 117, Post-immediate: 618 ± 126, Post-15 min: 614 ± 117, Post-30 min: 618 ± 126 *Dynamic test COP sway perimeter (mm) eyes open:* Static stretch Pre: 1189 ± 255, Post-immediate: 1188 ± 239, Post-15 min: 1189 ± 242, Post-30 min: 1188 ± 268 CG Pre: 1179 ± 277, Post-immediate: 1209 ± 272, Post-15 min: 1186 ± 259, Post-30 min: 1177 ± 254 *Dynamic test COP sway perimeter (mm) eyes closed:* Static stretch Pre: 1652 ± 358, Post-immediate: 1672 ± 400, Post-15 min: 1624 ± 370, Post-30 min: 1646 ± 376 CG Pre: 1664 ± 347, Post-immediate: 1666 ± 381, Post-15 min: 1654 ± 371, Post-30 min: 1639 ± 373
					*Dynamic test COP sway perimeter (mm) eyes open + foam pad:* Static stretch Pre: 1489 ± 306, Post-immediate: 1505 ± 308, Post-15 min: 1450 ± 287, Post-30 min: 1518 ± 325 CG Pre: 1506 ± 310, Post-immediate: 1491 ± 312, Post-15 min: 1496 ± 304, Post-30 min: 1499 ± 338 *Dynamic test COP sway perimeter (mm) eyes closed + foam pad:* Static stretch Pre: 2128 ± 379, Post-immediate: 2101 ± 441, Post-15 min: 2134 ± 414, Post-30 min: 2109 ± 411 CG Pre: 2112 ± 375, Post-immediate: 2139 ± 418, Post-15 min: 2064 ± 385, Post-30 min: 2118 ± 413 *Static test anteroposterior sway speed (cm/s) eyes open:* Static stretch Pre: 6.1 ± 0.8, Post-immediate: 5.6 ± 0.9, Post-15 min: 5.9 ± 0.8, Post-30 min: 5.9 ± 0.8 CG Pre: 5.8 ± 1.9, Post-immediate: 5.8 ± 0.9, Post-15 min: 5.8 ± 0.9, Post-30 min: 5.9 ± 0.9 *Static test anteroposterior sway speed (cm/s) eyes closed:* Static stretch Pre: 5.8 ± 0.8, Post-immediate: 5.4 ± 0.8, Post-15 min: 5.5 ± 0.8, Post-30 min: 5.8 ± 0.8 CG Pre: 6.4 ± 1.3, Post-immediate: 6.4 ± 1.3, Post-15 min: 6.3 ± 1.4, Post-30 min: 6.3 ± 1.3 *Static test anteroposterior sway speed (cm/s) eyes open + foam pad:* Static stretch Pre: 5.8 ± 0.8, Post-immediate: 5.5 ± 0.8, Post-15 min: 5.5 ± 0.8, Post-30 min: 5.8 ± 0.8 CG Pre: 5.81 ± 0.9, Post-immediate: 5.9 ± 0.8, Post-15 min: 5.7 ± 0.8, Post-30 min: 5.8 ± 0.9 *Static test anteroposterior sway speed (cm/s) eyes closed + foam pad:* Static stretch Pre: 6.2 ± 0.8, Post-immediate: 5.7 ± 0.8, Post-15 min: 5.9 ± 0.9, Post-30 min: 5.9 ± 0.8 CG Pre: 6.4 ± 0.9, Post-immediate: 6.2 ± 1.0, Post-15 min: 6.1 ± 0.9, Post-30 min: 6.2 ± 0.9 *Static test mediolateral sway speed (cm/s) eyes open:* Static stretch Pre: 3.8 ± 0.5, Post-immediate: 3.4 ± 0.5, Post-15 min: 3.5 ± 0.5, Post-30 min: 3.6 ± 0.5 CG Pre: 3.7 ± 0.5, Post-immediate: 3.8 ± 0.5, Post-15 min: 3.9 ± 0.6, Post-30 min: 3.8 ± 0.5 *Dynamic test mediolateral sway speed (cm/s) eyes closed:* Static stretch Pre: 4 ± 0.5, Post-immediate: 3.6 ± 0.4, Post-15 min: 3.8 ± 0.5, Post-30 min: 3.9 ± 0.5 CG Pre: 4 ± 0.6, Post-immediate: 4 ± 0.6, Post-15 min: 4 ± 0.6, Post-30 min: 4 ± 0.5 *Dynamic test mediolateral sway speed (cm/s) eyes open + foam pad:* Static stretch Pre: 3.7 ± 0.5, Post-immediate: 3.4 ± 0.5, Post-15 min: 3.5 ± 0.5, Post-30 min: 3.7 ± 0.5 CG Pre: 3.7 ± 0.5, Post-immediate: 3.7 ± 0.5, Post-15 min: 3.7 ± 0.6, Post-30 min: 3.7 ± 0.5 *Dynamic test mediolateral sway speed (cm/s) eyes closed + foam pad:* Static stretch Pre: 4.1 ± 0.6, Post-immediate: 3.4 ± 0.5, Post-15 min: 3.5 ± 0.5, Post-30 min: 3.7 ± 0.5 CG Pre: 4.1 ± 0.6, Post-immediate: 4.1 ± 0.7, Post-15 min: 4.1 ± 0.7, Post-30 min: 4.1 ± 0.5 No significant difference in overall balance control between the two groups.
Costa et al. (75)	Acute Passive Adults Healthy	*n* = 28 (*F* = 28), age: 24.7 ± 4.5 yrs., height: 160.7 ± 7.4 cm, weight: 60.6 ± 7.9 kg. Cross-over study with random sequence allocation (3 groups). Recreationally active.	Interventions: Static stretching and non-intervened CG. Muscle(s): Quadriceps, hamstrings, gastrocnemius, soleus. Static protocol 15 s: Unilateral stretch of the 4 muscles 3×15 s on both sides. Static protocol 45 s: Unilateral stretch of the 4 muscles 3×45 s on both sides. CG protocol: No intervention.	Overall stability index via Biodex Stability System (Biodex Medical Systems Inc., Shirley, NY, USA).	*Overall stability index (in °):* IG 15 s Pre: 3.7 ± 0.4, Post: 3.1 ± 0.3 IG 45 s Pre: 3.4 ± 0.3, Post: 3.7 ± 0.5 CG Pre: 3.2 ± 0.3, Post: 3.2 ± 0.2 Significant stability improvement only in 15-s stretching group compared to CG and 45-s stretching group.
Espí-López et al. (76)	Chronic Active Adults Healthy	*n* = 42 (no information on sex), age: 21.64 ± 3.81 yrs., Height: 171 ± 0.05 cm, Weight: 65.36 ± 11.9 kg. Parallel group design with random allocation (2 groups, PNF *n* = 20, manual therapy *n* = 22). Healthy, amateur field hockey players.	Interventions: PNF stretching and manual therapy (active CG). Muscle(s): Hamstrings, psoas, adductor, pyramidal, gluteus medius, quadriceps and anterior rectus. PNF protocol: No information on number of exercises. Each exercise 4 repetitions with 10 s stretch, 5 s submaximal voluntary contraction, 5 s relax, 15 s stretch. Manual therapy protocol: Treatment time-matched to PNF group with 7 exercises. Intervention period: 1 session/week for 3 weeks.	Y-balance Test (no information on equipment)	*YBT anterior reach (in cm)* PNF Pre: 62.6 ± 5.1, 1-week Post: 62.5 ± 5.0, 1-month Post: 61.5 ± 6.5 Manual therapy Pre 64.0 ± 6.8, 1-week Post: 64.1 ± 6.9, 1-month Post: 62.4 ± 4.3 *YBT posterolateral reach (in cm)* PNF Pre: 96.1 ± 10.6, 1-week Post: 102.8 ± 9.9, 1-month Post: 99.5 ± 10.7 Manual therapy Pre 89.4 ± 14.3, 1-week Post: 97.4 ± 8.4, 1-month Post: 99.4 ± 9.9 *YBT posteromedial reach (in cm)* PNF Pre: 90.6 ± 10.5, 1-week Post: 98.2 ± 8.0, 1-month Post: 97.5 ± 11.4 Manual therapy Pre 84.0 ± 14.7, 1-week Post: 91.7 ± 8.2, 1-month Post: 93.7 ± 10.4 Significant pre-post improvement in posterolateral and-medial YBT score for both PNF and manual therapy 1-week after the intervention end with the improvements lasting to 1-month after end of intervention in the manual therapy group only.
Fontana Carvalho et al. (77)	Chronic Active Adults Patients	*n* = 20 (*F* = 20), age = 29.5 ± 6 yrs., height: not reported, weight: not reported.	Interventions: Static passive stretching and lumbar stabilization exercise.	COP area via force plate (BIOMEC 400, EMG System do Basil)	*COP area (cm^2^) eyes open:* Stretching Pre: 2.8 ± 2.0, Post: 2.9 ± 1.8 Stabilization Pre: 3.7 ± 3.5, Post: 4.1 ± 4.3 *COP area (cm^2^) eyes closed:* Stretching Pre: 3.6 ± 1.9, Post: 3.3 ± 1.9 Stabilization Pre: 4.0 ± 4.2, Post: 3.2 ± 2.6
		Parallel group design with random allocation (2 groups each *n* = 10) Pregnant women with low-back pain, no info on training level.	Muscle(s): Tibial ischium, gluteus maximus, piriformis, paravertebral, quadratus lumborum, latissimus dorsi, scalene, trapezius. Stretching protocol: 8 exercises performed by physiotherapist (2–3× 15–20 s) Stabilization: 8 exercises on swiss ball (2–8 repetitions) Intervention period: 2 sessions/week for 6 weeks.		*Anteroposterior velocity (cm/s) eyes open:* Stretching Pre: 0.8 ± 0.2, Post: 0.2 ± 0.2 Stabilization Pre: 0.8 ± 0.2, Post: 0.2 ± 0.2 *Anteroposterior velocity (cm/s) eyes closed:* Stretching Pre: 4.8 ± 10.8, Post: 0.2 ± 0.2 Stabilization Pre: 1.1 ± 0.2, Post: 0.3 ± 0.3 *Mediolateral velocity (cm/s) with eyes open:* Stretching Pre: 0.2 ± 0.2, Post: 0.6 ± 0.1 Stabilization Pre: 0.2 ± 0.2, Post: 0.6 ± 0.1 *Mediolateral velocity (cm/s) eyes closed:* Stretching Pre: 0.6 ± 0.1, Post: 0.2 ± 0.1 Stabilization Pre: 0.6 ± 0.1, Post: 0.4 ± 0.2 Significant increase in postural stability for the velocity sway parameter in both groups. No difference between groups (reported).
Gajdosik et al. (78)	Chronic Passive Elderly Healthy	*n* = 19 (*F* = 19), age: 74.1 ± 3.9 yrs., height: 159.9 ± 5.3 cm, weight: 68.8 ± 8.6 kg. Parallel group design with random allocation (2 groups, IG *n* = 10, CG *n* = 9). Older women with limited dorsiflexion range of motion.	Interventions: Stretching and non-intervened CG. Muscle(s): Plantar flexors. Static stretch protocol: Unilateral stretch for both sides 10×15 s. CG protocol: No intervention. Intervention period: 3 sessions/week for 8 weeks.	Functional reach test via ruler	*Functional reach test (in cm)* IG Pre: 34.4 ± 4.6, Post: 34.7 ± 4.2 CG Pre: 31.7 ± 4.7, Post: 32.9 ± 4.4 No significant change/difference for either group.
Ghram et al. (52)	Acute Passive Adults Healthy	*n* = 14 (M = 14), age: 22.07 ± 2.16 yrs., height: 177 ± 7 cm, weight: 69.07 ± 10.88 kg. Cross-over study with random sequence allocation (2 groups). Recreationally active.	Interventions: PNF stretching and non-intervened CG. Muscle(s): Quadriceps, hamstrings, anterior tibialis and calf muscles. PNF protocol: Unilateral stretch for both sides each muscle and side 3 repetitions of 5 s isometric contraction +10 s of static stretching.	Bipedal static stance with eyes open and eyes closed on force platform PostureWin (Techno Concept, Cereste, France)	*Sway area (mm^2^) eyes open:* IG Pre: 240.8 ± 160.9, Post: 260.0 ± 89.2 CG Pre: 156.3 ± 81.9, Post: 202.3 ± 138.9 *Sway area (mm^2^) eyes closed:* IG Pre: 190.0 ± 112.2, Post: 213.4 ± 172.5 CG Pre: 135.3 ± 83.9, Post: 163.1 ± 86.5 *Sway velocity (mm/s) eyes open:* IG Pre: 8.3 ± 3.1, Post: 8.1 ± 1.7 CG Pre: 7.6 ± 1.7, Post: 7.7 ± 1.2 *Sway velocity (mm/s) eyes closed:* IG Pre: 8.4 ± 1.9, Post: 8.6 ± 2.9
			CG protocol: 10 min seated rest. Both groups performed a 5 min cycling warm-up.		CG Pre: 8.8 ± 2.2, Post: 8.8 ± 2.1 *CoP sway (in mm) in mediolateral (ML) direction eyes open:* IG Pre: 252.4 ± 117.3, Post: 235.2 ± 62.5 CG Pre: 206.0 ± 41.5, Post: 207.5 ± 42.5 *CoP sway (in mm) in mediolateral (ML) direction eyes closed:* IG Pre: 236.8 ± 70.5, Post: 224.0 ± 77.8 CG Pre: 221.6 ± 54.9, Post: 219.2 ± 62.7 *CoP sway (in mm) in anteroposterior (AP) direction eyes open:* IG Pre: 283.9 ± 89.4, Post: 289.9 ± 63.6 CG Pre: 288.5 ± 67.3, Post: 291.6 ± 44.0 *COP sway (in mm) in anteroposterior (AP) direction eyes closed:* IG Pre: 308.2 ± 62.4, Post: 332.9 ± 116.6 CG Pre: 346.7 ± 99.4, Post: 343.0 ± 84.9 Significant increase of sway area and anteroposterior sway in both conditions, but no difference between groups.
Ghram et al. (53)	Acute Passive Adults Healthy	*n* = 20 (M = 20), age: 21.3 ± 2.34 yrs., height: 177.7 ± 6.9 cm, weight: 69.2 ± 11.51 kg. Cross-over study with random sequence allocation (3 groups). Recreationally active.	Interventions: PNF-CR and PNF-CRAC and non-intervened CG. Muscle(s): Quadriceps, hamstrings, tibialis anterior, and triceps surae. PNF-CR protocol: Unilateral stretch for both sides each muscle and side 3 repetitions 5 s isometric contraction +5 s relaxation +5 s passive static stretch. PNF-CRAC protocol: Unilateral stretch for both sides each muscle and side 3 repetitions 5 s static stretch +5 s isometric contraction in agonist muscle +5 s isometric contraction in antagonist muscle. CG: 10 min rest. All groups performed a 5 min cycle warm-up.	Bipedal static stance with eyes open and eyes closed via force plate (PostureWin, Techno Concept, Cereste, France) and additional seesaw device (Stabilomètre, Techno Concept, Cereste, France).	*Sway (in mm) medio-lateral eyes closed* PNF-CR Pre: 754.4 ± 228.1, Post: 719.3 ± 219.3 PNF-CRAC Pre: 894.7 ± 271.9, Post: 596.5 ± 175.4 CG Pre: 894.7 ± 245.6, Post: 877.2 ± 280.7 *Sway (in mm) medio-lateral eyes open* PNF-CR Pre: 407.5 ± 105.7, Post: 392.5 ± 128.3 PNF-CRAC Pre: 415.1 ± 113.2, Post: 332.1 ± 45.3 CG Pre: 392.5 ± 101.9, Post: 392.5 ± 90.6 *Sway (in mm) antero-posterior eyes closed* PNF-CR Pre: 641.5 ± 196.2, Post: 603.8 ± 181.1 PNF-CRAC Pre: 664.2 ± 211.3, Post: 558.5 ± 181.1 CG Pre: 784.9 ± 241.5, Post: 679.2 ± 241.5 *Sway (in mm) antero-posterior eyes open* PNF-CR Pre: 362.3 ± 75.5, Post: 339.6 ± 113.2 PNF-CRAC Pre: 369.8 ± 128.3, Post: 290.6 ± 56.6 CG Pre: 384.9 ± 113.2, Post: 339.6 ± 75.5 Significant improvement of balance only in PNF-CRAC compared to CG. No significant difference between PNF groups.
Jouira et al. (54)	Acute Active Adults Patients	*n* = 12 (no information regarding sex), age: 24.5 ± 3.22 yrs., height: 165.7 ± 8.4 cm, weight: 61.5 ± 7.1 kg. Cross-over study without random allocation (2 groups). Athletes with intellectual disability.	Interventions: Dynamic stretching and plyometrics. Muscle(s): Hamstrings, iliopsoas, quadriceps, glutes, adductors. Dynamic stretching protocol: 6 exercises for 8×3 repetitions per exercise. Plyometrics protocol: 6 exercises for 8×3 repetitions per exercise. Both groups performed a 5 min jog prior to the intervention.	Star Excursion Balance Test	*SEBT anterior reach (in % of leg length)* Stretching Pre: 21.1 ± 6.8, Post: 84.02 ± 6.8, 15 min Post: 84.4 ± 6.4 Plyometrics Pre: 81.7 ± 6.7, Post: 81.1 ± 5.7, 15 min Post: 83.4 ± 5.7 *SEBT anterolateral reach (in % of leg length* Stretching Pre: 65.9 ± 8.3, Post: 67.0 ± 8.9, 15 min Post: 66.9 ± 8.6 Plyometrics Pre: 66.2 ± 7.6, Post: 65.9 ± 7.3, 15 min Post: 66.8 ± 6.9 *SEBT lateral reach (in % of leg length)* Stretching Pre: 70.3 ± 8.5, Post: 70.7 ± 8.4, 15 min Post: 71.1 ± 7.7 Plyometrics Pre: 70.6 ± 8.2, Post: 69.5 ± 9.2, 15 min Post: 70.7 ± 8.6 *SEBT posterolateral reach (in % of leg length)* Stretching Pre: 91.1 ± 8.3, Post: 94.4 ± 7.2, 15 min Post: 95.2 ± 7.8 Plyometrics Pre: 91.0 ± 8.6, Post: 90.6 ± 9.1, 15 min Post: 93.3 ± 8.3 *SEBT posterior reach (in % of leg length)* Stretching Pre: 99.0 ± 8.2, Post: 102.2 ± 7.8, 15 min Post: 102.8 ± 7.5 Plyometrics Pre: 98.6 ± 7.9, Post: 98.1 ± 8.4, 15 min Post: 100.8 ± 7.6 *SEBT posteromedial reach (in % of leg length)* Stretching Pre: 99.4 ± 7.5, Post: 101.7 ± 7.2, 15 min Post: 101.9 ± 7.4 Plyometrics Pre: 99.6 ± 7.8, Post: 98.3 ± 7.1, 15 min Post: 100.6 ± 7.9 *SEBT medial reach (in % of leg length)* Stretching Pre: 100.7 ± 7.4, Post: 102.9 ± 7.5, 15 min Post: 103.4 ± 7.1 Plyometrics Pre: 100.3 ± 6.7, Post: 99.5 ± 6.6, 15 min Post: 101.9 ± 6.7 *SEBT anteromedial reach (in % of leg length)* Stretching Pre: 87.8 ± 7.2, Post: 90.1 ± 7.3, 15 min Post: 90.6 ± 7.9 Plyometrics Pre: 87.9 ± 7.1, Post: 86.8 ± 6.7, 15 min Post: 89.4 ± 6.9 Significant increase for dynamic stretching compared to plyometrics.
Jung et al. (55)	Acute Passive Adults Healthy	*n* = 44 (*F* = 8, M = 36), age: 26.6 ± 2.2 yrs., height: 172.5 ± 7.2 cm, weight: 72 ± 13.8 kg. Parallel group design with random allocation (4 groups each *n* = 11). No info on training level.	Interventions: Static, dynamic, ballistic stretching and non-intervened CG. Muscle(s): Plantar flexors. Static protocol: Unilateral stretch of the dominant limb 4×45 s.	One-legged balance on AMTI AccuSway force plate (Advanced Mechanical Technology Inc., Watertown, MA, USA). No info for Y-balance equipment.	*Sway area (in mm^2^) with eyes open* Static stretch Pre: 6.2 ± 2.4, Post: 6.9 ± 2.5, 20 min follow-up: 7.2 ± 2.6 Dynamic stretch Pre: 7.4 ± 2.1, Post: 7.7 ± 2.6, 20 min follow-up: 8.9 ± 3.2 Ballistic stretch Pre: 8 ± 3.7, Post: 7.0 ± 1.8, 20 min follow-up: 6.8 ± 2.3 CG Pre: 8.2 ± 2.6, Post: 9.7 ± 8.0, 20 min follow-up: 9.4 ± 9.2 *Sway path length (in mm) with eyes open* Static stretch Pre: 43.0 ± 9.5, Post: 43.2 ± 6.5, 20 min follow-up: 42.6 ± 8.2 Dynamic stretch Pre: 50.7 ± 13.8, Post: 50.4 ± 10.0, 20 min follow-up: 49.5 ± 9.1 Ballistic stretch Pre: 45.2 ± 7.7, Post: 43.4 ± 9.8, 20 min follow-up: 42.1 ± 9.5 CG Pre: 52.4 ± 15.2, Post: 56.2 ± 15.8, 20 min follow-up: 51.9 ± 15.1 *Sway velocity (in mm/s) with eyes open*
			Dynamic protocol: Unilateral stretch of the dominant limb 4×45 s while repeatedly raising and lowering the heel (active movement once per second) Ballistic protocol: Unilateral stretch of the dominant limb 4×45 s with active movement as in dynamic but using a rebound at end ROM (active movement twice per second). CG protocol: No intervention.		Static stretch Pre: 4.3 ± 1.0, Post: 4.3 ± 0.7, 20 min follow-up: 4.3 ± 0.8 Dynamic stretch Pre: 5.1 ± 1.4, Post: 5.0 ± 1, 20 min follow-up: 5.0 ± 0.9 Ballistic stretch Pre: 4.5 ± 0.8, Post: 4.3 ± 1.0, 20 min follow-up: 4.2 ± 1.0 CG Pre: 5.2 ± 1.5, Post: 5.6 ± 1.6, 20 min follow-up: 5.2 ± 1.5 Significant increase in COP performance with eyes open for all three stretching interventions compared to CG. *Sway area (in mm^2^) with eyes closed* Static stretch Pre: 23.0 ± 7.3, Post: 18.9 ± 6.0, 20 min follow-up: 21.9 ± 7.0 Dynamic stretch Pre: 26.0 ± 11.0, Post: 21.1 ± 7.8, 20 min follow-up: 22.2 ± 5.7 Ballistic stretch Pre: 26.1 ± 7.6, Post: 22.4 ± 5.5, 20 min follow-up: 24.3 ± 6.3 CG Pre: 24.9 ± 7.5, Post: 23.3 ± 8.3, 20 min follow-up: 24.4 ± 7.9 *Path length (in mm) with eyes closed* Static stretch Pre: 83.5 ± 19.4, Post: 82.2 ± 18.6, 20 min follow-up: 83.7 ± 21.7 Dynamic stretch Pre: 97.2 ± 19.9, Post: 83.3 ± 14.4, 20 min follow-up: 88.2 ± 18.7 Ballistic stretch Pre: 88.3 ± 19.2, Post: 83.4 ± 17.8, 20 min follow-up: 83.8 ± 16.4 CG Pre: 78.9 ± 29.8, Post: 77.1 ± 27.8, 20 min follow-up: 75.9 ± 28.1 *Sway velocity (in mm/s) with eyes closed* Static stretch Pre: 8.4 ± 1.9, Post: 8.2 ± 1.9, 20 min follow-up: 8.4 ± 2.2 Dynamic stretch Pre: 9.7 ± 2.0, Post: 8.3 ± 1.4, 20 min follow-up: 8.8 ± 1.9 Ballistic stretch Pre: 8.8 ± 1.9, Post: 8.3 ± 1.8, 20 min follow-up: 8.4 ± 1.6 CG Pre: 9.1 ± 2.4, Post: 8.9 ± 2.0, 20 min follow-up: 8.8 ± 2.1 Significant increase in YBT performance for all three stretching interventions compared to CG. Significant increase in COP performance with eyes closed for all three stretching interventions compared to CG. *YBT anterior distance (in cm)* Static stretch Pre: 69.5 ± 7.1 Post: 72.1 ± 7.1, 20 min follow-up: 72.7 ± 8.2 Dynamic stretch Pre: 68.0 ± 6.3, Post: 70.6 ± 6.5, 20 min follow-up: 70.2 ± 6.8 Ballistic stretch Pre: 68.4 ± 4.5, Post: 70.9 ± 3.9, 20 min follow-up: 70.6 ± 3.7 CG Pre: 66.6 ± 6.3, Post: 67.0 ± 6.3, 20 min follow-up: 66.7 ± 6.0 *YBT posteromedial distance (in cm)* Static stretch Pre: 103.2 ± 11.5, Post: 106.9 ± 11.0, 20 min follow-up: 106.3 ± 12.1 Dynamic stretch Pre: 98.6 ± 7.1, Post: 103.8 ± 8.6, 20 min follow-up: 104.6 ± 7.7 Ballistic stretch Pre: 99.6 ± 8.2, Post: 105.3 ± 8, 20 min follow-up: 107.1 ± 7.0 CG Pre: 104.1 ± 11.7, Post: 104.1 ± 11.6, 20 min follow-up: 104.5 ± 10.5 *YBT posterolateral distance (in cm)* Static stretch Pre: 99.3 ± 12.6, Post: 103.4 ± 13.2, 20 min follow-up: 103.0 ± 13.9
					Dynamic stretch Pre: 94.1 ± 10.7, Post: 100.1 ± 10.9, 20 min follow-up: 100.9 ± 10.7 Ballistic stretch Pre: 93.6 ± 9.3, Post: 97.8 ± 8.8, 20 min follow-up: 98.1 ± 8.0 CG Pre: 98.1 ± 15.4, Post: 98.6 ± 14.0, 20 min follow-up: 99.7 ± 14.1
Kim et al. (56)	Acute Active Adults Healthy	*n* = 22 (*F* = 12, M = 10), age: no detailed information, height: 166.11 ± 0.84 cm, weight: 59.1 ± 0.91 kg. Cross-over study without random sequence allocation (3 groups). Healthy students, no info on training level.	Interventions: Static stretching, plyometrics (active CG) and treadmill walking (active CG). Muscle(s): Quadriceps, hamstrings, gastrocnemius and soleus. Static stretching protocol: Bilateral stretch 3×45 s per muscle group. Plyometric protocol: 4 exercises for 5×45 s per exercise to metronome speed of 100 bpm. Treadmill protocol: Walking at a speed of 1.2 m/s for 16 min.	Limits of stability via BioRescue (RM Ingénierie, Rodez, France).	*Limits of stability (no info on units)* Static stretch Pre: 14,936.6 ± 3,816, Post: 15,292.1 ± 4,305.2, 20 min follow-up: 15,833.2 ± 3,977.9 Plyometric Pre: 14,948.1 ± 5,275.8, Post: 13,545.9 ± 5,467.8, 20 min follow-up: 15,231.9 ± 5,482.4 Treadmill Pre: 15,528.6 ± 4,075.3, Post: 14,568.9 ± 3,962.2, 20 min follow-up: 14,707.3 ± 3,940.8 No significant difference between the three conditions.
Leblebici et al. (57)	Acute Passive Adults Healthy	n = 12 (M = 12), age: 19.67 ± 2.23 yrs., height: 172.33 ± 4.52 cm, weight: 67.56 ± 8.92 kg. Cross-over study with random sequence allocation (4 groups). Active athletes, no further info.	Interventions: Static stretching, dynamic stretching, PNF stretching and non-intervened CG. Muscle(s): Quadriceps, hamstrings and plantar flexors. Static stretching protocol: Unilateral stretching of both legs for 3×30 s for each muscle. Dynamic stretching protocol: Unilateral stretching of both legs with 1 exercise per muscle with 3 sets each consisting of 5 slow and 10 fast repetitions. PNF protocol: Unilateral stretching of both legs for 3 sets with 10 s of stretch, 6 s maximal isometric contraction and 14 s of passive stretch. CG protocol: 5 min of rest.	Overall stability index via Biodex (Biodex Balance System, Inc., EN) using level 3.	*Overall stability index (no info on units)* Static stretch Pre: 0.8 ± 0.295, Post: 0.9 ± 0.204 Dynamic stretch Pre: 0.842 ± 0.271, Post: 0.95 ± 0.329 PNF stretch Pre: 0.883 ± 0.369, Post: 0.808 ± 0.332 CG Pre: 0.775 ± 0.29, Post: 0.842 ± 0.235 No significant difference between the four conditions.
Lim et al. (58)	Acute Passive Adults Healthy	*n* = 48 (M = 48), age: 22.71 ± 2.25 yrs., height: 173.31 ± 4.94 cm, weight: 68.52 ± 9.43 kg. Parallel group design with random allocation (3 groups each *n* = 16). Healthy adults with hamstring tightness, no info on training level.	Interventions: Static stretching, PNF stretching and non-intervened CG. Muscle(s): Hamstrings. Static stretching protocol: Unilateral stretch for 1×30 s. PNF protocol: Unilateral CR application 1 set of 3×6 s maximal voluntary contraction in lengthened position with 5 s rest between contractions. CG protocol: 30 s rest.	Postural sway via force plate (PDM, Multifunction Force Measuring Plate, Zebris, Germany, 2004).	*Mediolateral postural sway (no info on units)* Static stretch Pre: 137.7 ± 41.6, Post: 122.0 ± 38.1 PNF stretch Pre: 142.5 ± 42.6, Post: 130.1 ± 36.7 CG Pre: 151.7 ± 40.3, Post: 157.1 ± 47.9 *Anteroposterior postural sway (no info on units)* Static stretch Pre: 121.9 ± 35, Post: 12 ± 27.1 PNF stretch Pre: 115.3 ± 20.6, Post: 114.2 ± 12.6 CG Pre: 111.2 ± 26.9, Post: 116.8 ± 24.7 No significant difference between the three conditions.
Lima et al. (59)	Acute Passive Adults Healthy	*n* = 14 (M = 7, *F* = 7), age: 23.5 ± 3 yrs., height: 169 ± 5 cm, weight: 67.5 ± 7 kg. Intra-individual control leg. Non-trained individuals.	Interventions: Static stretching and non-intervened CG. Muscle(s): Plantar flexors. Static stretching protocol: Unilateral stretch 6×45 s. CG protocol: No intervention.	Single leg postural sway via force plate (Kistler model 9286A, Winterthur, Switzerland).	*COP sway area (in mm^2^)* Static stretch Pre: 831.6 ± 368.4, Post: 1094.7 ± 421.1 CG Pre: 743.9 ± 298.3, Post: 901.8 ± 333.3 *COP sway anteroposterior speed (in mm/s)* Static stretch Pre: 28.1 ± 5.7, Post: 29.9 ± 10.3 CG Pre: 26.2 ± 6.3, Post: 28.5 ± 9.8 *COP sway mediolateral speed (in mm/s)* Static stretch Pre: 23.2 ± 5.3, Post: 26.6 ± 14.6 CG Pre: 21.1 ± 3.8, Post: 21.9 ± 5.1 *COP sway anteroposterior frequency (in Hz)* Static stretch Pre: 1.06 ± 0.24, Post: 0.87 ± 0.16 CG Pre: 1.1 ± 0.28, Post: 0.82 ± 0.18 *COP sway mediolateral frequency (in Hz)* Static stretch Pre: 0.65 ± 0.23, Post: 0.62 ± 0.25 CG Pre: 0.51 ± 0.19, Post: 0.74 ± 0.28 Sway was significantly higher following the static stretch condition compared to the intra-individual CG.
Mel’nikov et al. (60)	Chronic Passive Adults Healthy	*n* = 28 (*F* = 28), age: 18–21 yrs., height: 164.3 ± 5.7 cm, weight: 58.4 ± 7.8 kg. Parallel group design without random allocation (2 groups each *n* = 14).	Interventions: Stretching and non-intervened CG. Muscle(s): Lower body. Stretching protocol: 15 min of general warm-up (running and jumping) plus 20 min of dynamic stretching in motion, 20 min of dynamic stretching	Single leg COP with open- and closed-eyes via Neurocor Trast-M stabiloplatform (Russia)	*COP sway oscillations in sagittal plane with eyes open (in mm)* Stretching Pre: 7.5 ± 2.4, Post: 5.3 ± 0.8 CG Pre: 6.2 ± 1.8, Post: 6.2 ± 1.6 *COP sway oscillations in frontal plane with eyes open (in mm)* Stretching Pre: 4.6 ± 1.3, Post: 4.4 ± 0.7 CG Pre: 3.9 ± 0.7, Post: 4.3 ± 1 *COP sway average linear velocity in sagittal plane with eyes open (in mm/s)* Stretching Pre: 19.7 ± 7, Post: 18.7 ± 5.8
		Physically active, no further info on training level.	in place and 20 min of static stretching in place. CG: No intervention, continuing normal lifestyle. Intervention period: 3 sessions/week for 10 weeks.		CG Pre: 18.3 ± 6.2, Post: 18.6 ± 4.5 *COP sway average linear velocity in frontal plane with eyes open (in mm/s)* Stretching Pre: 20 ± 5.8, Post: 21.7 ± 5 CG Pre: 18.9 ± 5.7, Post: 19.9 ± 4.3 *COP sway area with eyes open (in mm^2^)* Stretching Pre: 412.9 ± 295.9, Post: 258.3 ± 70.6 CG Pre: 269 ± 88.2, Post: 317.6 ± 156 *COP sway oscillations in sagittal plane with eyes closed (in mm)* Stretching Pre: 10.8 ± 2.8, Post: 8.9 ± 1.9 CG Pre: 9.7 ± 2.5, Post: 8.6 ± 2.7 *COP sway oscillations in frontal plane with eyes closed (in mm)* Stretching Pre: 8.9 ± 1.9, Post: 8 ± 1 CG Pre: 9.9 ± 4.3, Post: 7.8 ± 1.9 *COP sway average linear velocity in sagittal plane with eyes closed (in mm/s)* Stretching Pre: 42.5 ± 12.3, Post: 39.6 ± 11.2 CG Pre: 44.8 ± 15, Post: 40.8 ± 17.1 *COP sway average linear velocity in frontal plane with eyes closed (in mm/s)* Stretching Pre: 41.7 ± 9, Post: 41.8 ± 9 CG Pre: 40.7 ± 10.9, Post: 40.3 ± 9.6 *COP sway area with eyes closed (in mm^2^)* Stretching Pre: 1110.4 ± 448, Post: 822.4 ± 249.1 CG Pre: 1115.1 ± 881.2, Post: 782.5 ± 432.1 Significant increase in stability following the stretching group compared to CG only for the sagittal plane under open-eyes condition.
Oba et al. (61)	Acute Passive Adults Healthy	*n* = 26 (M = 26), age: 21.4 ± 1.2 yrs., height: 171.5 ± 5.6 cm, weight: 63.9 ± 7.8 kg. Cross-over study with random allocation (2 groups). No info on training level.	Interventions: Static stretching and non-intervened CG. Muscle(s): Plantar flexors. Stretching protocol: Bilateral stretch 5×60 s. CG protocol: No intervention.	COP in double-leg stance with eyes open via force plate (FDM-S ver. 1.2.0, Zebris Medical GmbH, Germany)	*COP sway area during static standing (in mm^2^)* Static stretch Pre: 92.7 ± 13.2, Post: 105.9 ± 11.4 CG Pre: 92.8 ± 48.2, Post: 89.3 ± 47.5 *COP sway mean mediolateral position during static standing (in mm)* Static stretch Pre: −2.7 ± 6.9, Post: −3.5 ± 6.4 CG Pre: −1.5 ± 6.5, Post: −0.74 ± 6.9 *COP sway mean velocity during static standing (in mm/s)* Static stretch Pre: 6.7 ± 1.6, Post: 7.7 ± 2 CG Pre: 6.9 ± 1.3, Post: 7.1 ± 1.2 *COP sway area during maximum forward leaning (in mm^2^)* Static stretch Pre: 213.9 ± 88.3, Post: 242.3 ± 92.7 CG Pre: 214.9 ± 74.4, Post: 247 ± 110.8
					*COP sway mean mediolateral position during maximum forward leaning (in mm)* Static stretch Pre: 0.05 ± 9.5, Post: −0.86 ± 9.3 CG Pre: −0.86 ± 8.1, Post: 1.1 ± 7.8 *COP sway mean velocity during maximum forward leaning (in mm/s)* Static stretch Pre: 13.9 ± 3, Post: 15.9 ± 3.6 CG Pre: 14.1 ± 3.3, Post: 14 ± 3.1 Significant increase in velocity and anteroposterior position during double-leg standing and maximum forward lean for the stretch group compared to CG. No significant difference between groups for COP area.
Oba et al. (63)	Acute Passive Adults Healthy	*n* = 15 (*F* = 3, M = 12), age: 23.9 ± 2.4 yrs., height: 172.4 ± 8.1 cm., weight: 62.5 ± 7.6 kg. Cross-over study with random allocation (3 groups). Partly recreationally active and partly no regular sports activities.	Interventions: Static stretching, dynamic stretching and non-intervened CG. Muscle(s): Plantar flexors. Static stretching protocol: Unilateral stretch 4×30 s of dominant leg. Dynamic stretching protocol: 15 maximal dorsi and plantar flexion repetitions without bouncing within 30 s. 4×30 s on dominant side. CG protocol: No intervention (standing).	Single-leg stance with eyes open on force plate (FDM-S ver. 1.2.0, Zebris Medical GmbH, Germany)	*COP sway area (mm^2^)* Static Stretch Pre: 457.2 ± 108.3, Post: 477.8 ± 106.1 Dynamic Stretch Pre: 498.6 ± 148.3, Post: 393.3 ± 101.1 CG Pre: 477.0 ± 128.8, Post: 497.6 ± 165.8 *COP sway velocity (mm/s):* Static Stretch Pre: 31.2 ± 4.2, Post: 30.7 ± 5.8 Dynamic Stretch Pre: 33.8 ± 7.6, Post: 29.8 ± 6.5 CG Pre: 33.3 ± 7.2, Post: 32.0 ± 7.3 *COP sway anteroposterior range (mm):* Static Stretch Pre: 25.4 ± 3.1, Post: 25.3 ± 3.2 Dynamic Stretch Pre: 26.1 ± 5.5, Post: 23.6 ± 3.6 CG Pre: 26.1 ± 4.6, Post: 25.9 ± 4.6 *COP sway mediolateral range (mm):* Static Stretch Pre: 20.7 ± 3.3, Post: 21.1 ± 2.5 Dynamic Stretch Pre: 21.5 ± 4.1, Post: 19.0 ± 2.5 CG Pre: 20.8 ± 2.9, Post: 21.6 ± 3.7 Significant pre-post decrease in dynamic stretch for COP area, velocity and mediolateral range compared to CG and static stretch group.
Park et al. (64)	Chronic Active Adults Patients	*n* = 20 (*F* = 12, M = 8), age: 58.85 ± 6.5 yrs., height: 165.05 ± 6.1 cm, weight: 64.8 ± 8.7 kg. Parallel group design with random allocation (2 groups each *n* = 10). Patients with chronic stroke.	Interventions: Static stretching and mobilization with movement Muscle(s): Calf muscle. Static stretching protocol: 3 sets of 10×30 s. Mobilization protocol: Lunge with passive stabilization of ankle by therapist. 3 sets of 10×30 s.	Biodex Balance System SD (BBS, Shirley, NY, USA)	*Static balance ability (score):* Static stretch Pre: 0.9 ± 0.29, Post: 0.72 ± 0.21 Mobilization Pre: 1.06 ± 0.41, Post: 0.47 ± 0.13 Significant pre-post balance improvement only in mobilization group.
			Intervention period: 3 sessions/week for 4 weeks.		
Ryan et al. (65)	Acute Passive Adults Healthy	*n* = 30 (*F* = 15, M = 15), age: 25.17 ± 5.4 yrs., height: 173.76 ± 8.2 cm, weight: 72.03 ± 14.87 kg. Parallel group design without random allocation (3 groups each *n* = 10). Healthy individuals.	Interventions: PNF stretching and Warm-up+PNF stretching and non-intervened CG Muscle(s): Quadriceps, hamstrings, iliopsoas, plantar flexors. PNF stretching protocol: Passive initial stretch +7 s agonist isometric contraction +4 s antagonist contraction. 4× for quadriceps, hamstrings and iliopsoas; 3× for plantar flexors. Warm-up+PNF protocol: 6 min treadmill jogging (65% of maximum heart rate reserve) + PNF stretching protocol. CG: 12 min seated rest.	Overall stability index via Biodex Balance System (Biodex Medical Systems, Inc., Shirley, NY, USA)	*Overall stability index:* PNF Pre: 4.03 ± 2.58, Post: 3.45 ± 2.51 Warm-up+PNF Pre: 3.90 ± 2.62, Post: 3.48 ± 2.26 CG Pre: 3.90 ± 2.72, Post: 3.65 ± 2.63 *Anteroposterior stability index:* PNF Pre: 3.26 ± 2.02, Post: 2.81 ± 1.92 Warm-up+PNF Pre: 3.15 ± 2.14, Post: 2.77 ± 1.63 CG Pre: 3.21 ± 2.33, Post: 2.80 ± 2.06 *Mediolateral stability index:* PNF Pre: 2.53 ± 1.65, Post: 1.95 ± 0.81 Warm-up+PNF Pre: 2.51 ± 1.53, Post: 1.84 ± 0.83 CG Pre: 2.44 ± 1.60, Post: 2.40 ± 1.69 Mediolateral stability significantly improved in PNF and Warm-up+PNF compared to CG. No significant difference between PNF vs. Warm-up+PNF.
Sakai et al. (66)	Chronic Passive Adults Healthy	*n* = 18 (M = 18), age: 22.5 ± 1.4 yrs., height: 171.9 ± 5.8 cm, weight: 63.9 ± 8.6 kg. Parallel group design with random allocation (2 groups, IG *n* = 9, CG *n* = 9). No competitive athletes, but engaged in systematic resistance training and stretching programs.	Interventions: Cyclic stretching and non-intervened CG. Muscle(s): Plantar flexors. Stretching protocol: 2 min cyclic stretching of plantar flexor muscles CG protocol: No intervention (standing on the device). Intervention period: 5 sessions/week for 4 weeks.	Postural stability via force plate (Myotest SA, Sion, Switzerland)	*Dynamic postural stability index:* CS Pre:0.31 ± 0.01, Post: 0.28 ± 0.06 CG Pre: 0.29 ± 0.06, Post: 0.31 ± 0.06 *Mediolateral stability index:* CS Pre: 0.03 ± 0.01, Post: 0.02 ± 0.00 CG Pre: 0.03 ± 0.01, Post: 0.03 ± 0.01 *Anteroposterior stability index:* CS Pre: 0.14 ± 0.01, Post: 0.12 ± 0.01 CG Pre: 0.13 ± 0.01, Post: 0.13 ± 0.01 *Vertical stability index:* CS Pre: 0.28 ± 0.02, Post: 0.25 ± 0.06 CG Pre: 0.26 ± 0.07, Post: 0.27 ± 0.06 No significantly different change between groups.
Szafraniec et al. (67)	Acute Passive Adults Healthy	*n* = 45 (*F* = 29, M = 16), age: 20.9 ± 1.3 yrs., height: 172 ± 5.9 cm, weight: 70.6 ± 9.5 kg. Parallel group design with random allocation (2 groups, PNF *n* = 31, CG *n* = 14). No info on training level.	Interventions: PNF stretching and non-intervened CG. Muscle(s): Hip adductors and abductors. PNF protocol: Unilateral stretch of both limbs each 3 sets of 10 s 50% maximal voluntary contraction in lengthened position, 5 s relaxation, 5 s stretch. CG protocol: 5 min seated rest.	Mediolateral sway with open eyes via Libra stabilometric platform (Libra, EasyTech, Salerno, Italy).	*Total area of sway (in °s)* PNF Pre: 69.18 ± 16.45, Post: 56.42 ± 9.69 CG Pre: 71.16 ± 12.96, Post: 70.93 ± 16.2 *External area of sway (in °s)* PNF Pre: 4.61 ± 5.49, Post: 1.03 ± 1.3 CG Pre: 5.86 ± 5.94, Post: 4.11 ± 5.09 *External time (s)* PNF Pre: 2.57 ± 1.97, Post: 1.06 ± 1.15 CG Pre: 2.71 ± 1.64, Post: 2.71 ± 2.12 *Global index* PNF Pre: 3.45 ± 1.34, Post: 2.42 ± 0.73 CG Pre: 3.62 ± 0.98, Post: 3.57 ± 1.38 Significant decrease in sway for the stretch group compared to CG.
Thomas et al. (68)	Acute Active & passive Adults Healthy	*n* = 32 (*F* = 13, M = 19), age: 25.3 ± 5.6 yrs., height: 172.0 ± 8.8 cm, weight: 68.77 ± 12.5 kg. Cross-over study with random allocation (3 groups). Healthy participants, competitive athletes were excluded.	Interventions: Static stretching, PNF stretching, positional transversal release and non-intervened CG. Muscle(s): Hamstrings. Static stretch protocol: 8×30 s passive trunk flexion in seated position. PNF protocol: 8 repetitions of 10 s stretch, 6 s agonist isometric contraction, 4 s post-isometric relaxation. Positional transversal release protocol: 1 to 2 mechanical stimulations of the proximal insertion of the hamstring muscles. CG protocol: 15 min sitting.	Y-balance test kit (Functional Movement Systems, Chatham, USA)	*YBT composite reach (in % of leg length) right leg:* Static Stretch Pre: 97.1 ± 6.6, Post: 97.4 ± 6.0, 15-min follow-up: 97.4 ± 6.5 PNF Pre: 96.9 ± 6.5, Post: 97.9 ± 7.0, 15-min follow-up: 98.0 ± 7.5 Positional transversal release Pre: 97.0 ± 6.7, Post: 97.6 ± 6.8, 15-min follow-up: 98.0 ± 6.8 CG Pre: 93.2 ± 7.5, Post: 94.9 ± 7.7, 15-min follow-up: 96.0 ± 7.6 *YBT composite reach (in % of leg length) left leg:* Static Stretch Pre: 96.5 ± 6.7, Post: 67.3 ± 6.7, 15-min follow-up: 67.4 ± 6.5 PNF Pre: 96.1 ± 6.9, Post: 97.0 ± 7.1, 15-min follow-up: 97.4 ± 6.8 Positional transversal release Pre: 97.0 ± 6.6, Post: 97.2 ± 6.9, 15-min follow-up: 97.4 ± 7.1 CG Pre: 92.5 ± 9.2, Post: 94.4 ± 7.0, 15-min follow-up: 95.4 ± 7.8 No significant difference within and between groups.
Todde et al. (69)	Chronic Passive Adults Healthy	*n* = 36 (*F* = 10, M = 26), age: 24.27 ± 4.87 yrs., height: 169.25 ± 10.97 cm, weight: no info.	Interventions: Warm-up (only) and Warm-up + static stretch and Warm-Up + PNF stretch. Muscle(s): Hamstrings, quadriceps, iliopsoas, and calf.	Ellipse area via Baropodometry (Zebris treadmill system)	*Ellipse area (mm^2^)* Static stretch Pre: 111.3 ± 35.8, Post: 135.0 ± 32.3 PNF Pre: 119.1 ± 23.0, Post: 88.2 ± 19.8 CG Pre: 118.0 ± 34.6, Post: 115.0 ± 26.9 Significant improvement in PNF group and significant decrease in static stretching compared to CG.
		Parallel group design with random group allocation (3 groups each *n* = 12). Sport science students.	Warm-up protocol: Treadmill running at 10 km/h, 1% of slope for 10 min. Warm-up + static stretching protocol: Warm-up protocol plus unilateral stretch for the 4 muscles on both sides for 4×30 s. Warm-up + PNF protocol: Warm-up protocol plus stretching agonist 5–10 s, relaxation of 3–5 s, contraction of antagonist for 5–10s, passive stretch of agonist for 20–30s, relaxation of 30 s. 4 repetitions per muscle per side. Intervention period: 3 sessions/week for 8 weeks.		
Tollár et al. (70)	Chronic Active & passive Adults Patients	*n* = 68 (*F* = 61, M = 7), age: 47.0 ± 5.95 yrs., height: 170.6 ± 5.3 cm, weight: 58.3 ± 8.27 kg. Parallel group design with random allocation (5 groups, PNF *n* = 14, exergaming *n* = 14, balance *n* = 14, cycling *n* = 14, CG *n* = 12) Patients with multiple sclerosis.	Interventions: PNF stretching, exergaming, balance, cycling and CG. Muscle(s): Upper and lower extremities. PNF stretching protocol: 10 min warm-up, 40 min PNF intervention by physical therapist (10 min dynamic and stabilizing reversals and rhythmic stabilization, 20 min of PNF using the Contract-Relax and Hold-Relax method), 10 min cool-down.	Sway in narrow and wide stance on force plate (Posture Evaluation Platform, Med-Eval Co., Budapest, Hungary)	*COP sway (cm) wide stance, eyes open:* PNF Change: −1.8 ± 3.99 Exergaming Change: −5.5 ± 4.20 Balance Change: −2.4 ± 3.62 Cycling Change: −1.7 ± 3.64 CG Change: 0.4 ± 3.34 *COP sway (cm) wide stance, eyes closed:* PNF Change: −0.8 ± 3.01 Exergaming Change: −2.0 ± 3.51 Balance Change: −1.5 ± 3.14 Cycling Change: −0.9 ± 3.36 CG Change: −1.0 ± 3.55 *COP sway (cm) narrow stance, eyes open:* PNF Change: −0.9 ± 5.83 Exergaming Change: −3.9 ± 7.41 Balance Change: −2.1 ± 7.95 Cycling Change: −2.2 ± 5.63 CG Change: −0.5 ± 8.18
			Exergame protocol: 10 min warm-up, 40 min sensorimotor and visuomotor agility training (Xbox 360 core system, Kinect Adventures video games, Microsoft Co., Redmond, WA), 10 min cool-down. Balance protocol: 10 min warm-up, 40 min dynamic and static balance stepping exercises performed in multiple directions, 10 min cool-down. Cycling protocol: 10 min warm-up, 40 min “spinning class,” 10 min cool-down. CG: Continue standard physical therapy and habitual activity. Intervention period: 5 sessions/week for 5 weeks.		*COP sway (cm) narrow stance, eyes closed:* PNF Change: −0.9 ± 4.04 Exergaming Change: −2.9 ± 5.20 Balance Change: −1.6 ± 4.85 Cycling Change: −1.7 ± 4.90 CG Change: 0.5 ± 5.22 No significant improvement for PNF compared to CG. Exergaming and balance groups significantly reduced sway compared to PNF, cycling and CG.
Wallmann et al. (71)	Acute Passive Adults & elderly Healthy	*n* = 48 (*F* = 29, M = 19), height: not reported, weight: not reported. Repeated measures design without randomization (1. CG, 2. Stretching). Two age groups included: adults (*n* = 30, age: 25.8 ± 2.3 yrs.) and elderly (*n* = 18, age: 72 ± 7 yrs.).	Interventions: Stretching (Pre-Test 1 vs. Post-Test) and non-intervened CG (Pre-Test 1 vs. Pre-Test 2) Muscle(s): Gastrocnemius. Stretching protocol: 3×30 s bilateral static stretch of gastrocnemius muscle. CG protocol: 2 min sitting.	Limits of stability via NeuroCom SMART Balance Master (NeuroCom International, Inc., 9,750 SE Lawnfield Road, Clackamas, OR 97015)	*Movement velocity (°/s), young participants:* Stretch Pre: 4.57 ± 1.26, Post: 5.32 ± 1.29 CG Pre: 4.57 ± 1.26, Post: 5.19 ± 1.21 *Movement velocity (°/s), old participants:* Stretch Pre: 2.91 ± 0.73, Post: 3.29 ± 0.82 CG Pre: 2.91 ± 0.73, Post: 3.17 ± 0.81 *Endpoint excursion (% of total LOS distance), young participants:* Stretch Pre: 75.37 ± 7.33, Post: 78.80 ± 7.37 CG Pre: 75.37 ± 7.33, Post: 77.63 ± 6.95 *Endpoint excursion (% of total LOS distance), old participants:* Stretch Pre: 56.28 ± 10.36, Post: 61.78 ± 12.97 CG Pre: 56.28 ± 10.36, Post: 58.67 ± 9.06 *Maximum excursion (no unit info), young participants:* Stretch Pre: 85.37 ± 6.85, Post: 87.27 ± 6.06 CG Pre: 85.37 ± 6.85, Post: 86.27 ± 5.85
					*Maximum excursion (no unit info), old participants:* Stretch Pre: 71.72 ± 11.07, Post: 74.72 ± 11.59 CG Pre: 71.72 ± 11.07, Post: 73.72 ± 10.33 *Directional control (no unit info), young participants:* Stretch Pre: 83.30 ± 4.90, Post: 84.60 ± 3.81 CG Pre: 83.30 ± 4.90, Post: 83.77 ± 3.78 *Directional control (no unit info), old participants:* Stretch Pre: 73.61 ± 12.04, Post: 78.22 ± 9.18 CG Pre: 73.61 ± 12.04, Post: 78.22 ± 8.31 No effect of stretching on limits of stability.

bpm, Beats per minute; BS, Ballistic stretching; CG, Control group; cm, Centimeter; COP, Center of pressure; CR, Contract relax; CRAC, Contract relax antagonist contract; DS, Dynamic stretching; f, female; Hz, Hertz; IG, Intervention group; LOS, Limits of stability; m, male; mm, Millimeter; n, Number; PNF, Proprioceptive neuromuscular facilitation; s, Seconds; SEBT, Star Excursion balance test; SS, Static stretch; TENS, Transcutaneous electrical nerve stimulation; YBT, Y-balance test. *Tags for the time period acute/chronic, comparison group active/passive, age group adults/elderly, health status healthy/patients. cm; Centimeter; kg, Kilogram; M±SD, Mean ± Standard deviation; Pre; Pre-test; Post, Post-test; yrs., Years.

### 3.1 Methodological quality and risk of bias

With a PEDro score (M ± SD) of 4.2 ± 1.8 out of 10 (range: 1 to 8 points), risk of bias was rated as fair. When differentiating for acute and chronic studies, the average score is 3.5 and 5.4, respectively, indicating that acute studies had a lower quality. While the classification fair fits the chronic studies’ quality assessment, the acute studies’ overall quality must be rated as poor. Overall, all studies provided both point measures and measures of variability (29/29), while almost all studies reported statistical between-group comparisons (26/29) and random group (for parallel group designs) or intervention sequence (for cross-over designs) allocation (22/29). While blinding of the participants and therapists was never achieved, investigator-blinding was performed in seven studies. Only seven studies concealed the allocation and only five studies adhered to the application of the intention-to-treat principle. In 13 out of 29 studies the groups were not considered similar at baseline regarding the most important prognostic factors. Only ten studies specifically stated both, the number of subjects in both pre- and post-testing and collected data from at least 85% of the initially included subjects (see Appendix Table 1).

### 3.2 Chronic stretching effects

Seven studies investigated chronic effects of stretching compared to passive controls, whereby one study (70) incorporated both passive and active controls. For the passive control comparisons, six studies (51, 60, 62, 66, 69, 70) incorporated young adults (18–65 yrs. of age). There was only one study (78) that investigated the chronic effects of stretching on older adults’ balance performance, who performed stretching for eight weeks, thrice per week with 150 s of stretch per session and found no change. Furthermore, two out of the seven studies investigated patients post ankle sprain (51) or with multiple sclerosis (70). Sensitivity analysis excluding the patient populations (due to the possibility of different neuromuscular responses for interventions in patients settings (79, 80)) resulted in non-significant results (*p* = 0.094–0.241).

**Figure 3:**
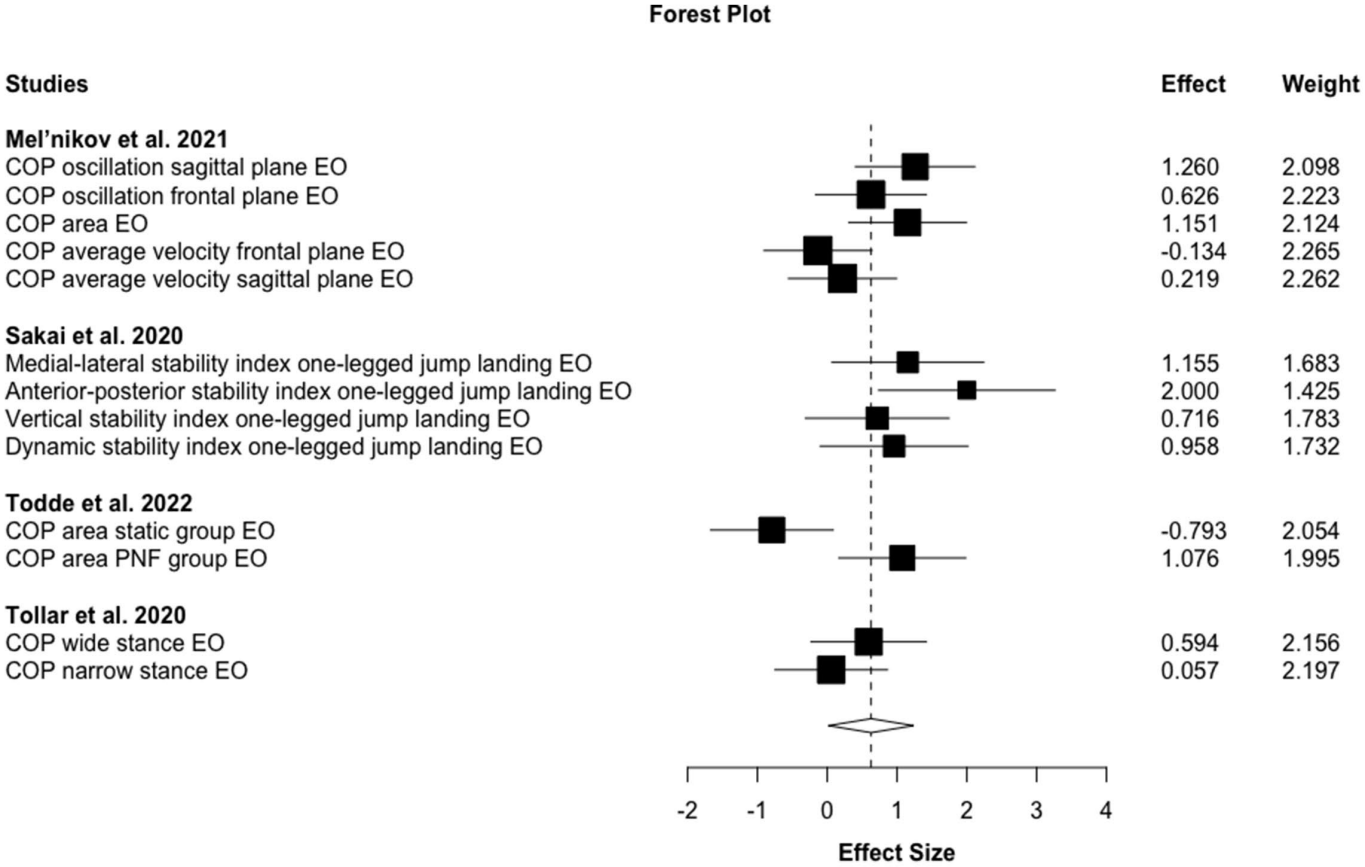
Forest plot for chronic stretching effects on sway balance against passive control conditions.

Out of the seven studies, four investigated static standing sway/COP parameters with eyes open of which three (60, 66, 69) found a positive effect, one (70) no change and one (69) a negative effect. Hereby, Todde et al. (69) found both a large increase and large decrease for two different subgroups following different stretching interventions. Effect size pooling exhibit a moderate magnitude improvement (ES: 0.63 95%CI 0.02–1.24, *p* = 0.047, τ^2^ = 0.12) (see Figure 3). Sensitivity analysis (excluding stability index outcomes to account for the possible impact of an index ceiling effect (81–84)) resulted in small magnitude effects, however, non-significant for the same outcomes (ES: 0.44 95%CI -0.18–1.06, *p* = 0.091, τ^2^ = 0) (see Table 2).

**Table 2:** Meta-analytic calculations for chronic and acute effects.

Parameter	ES (95% CI)	*p*-value	Heterogeneity (τ^2^)	N of studies/outcomes
Chronic effects vs. passive controls				
COP/Sway EO (without stability index outcomes)	0.44 (−0.18–1.06)	0.091	0	3/9
COP/Sway EO (without stability index outcomes) without patients	0.48 (−1.94–2.90)	0.241	0	2/7
COP/Sway EO (with stability index outcomes)	0.63 (0.02–1.24)	0.047	0.12	4/13
COP/Sway EO (with stability index outcomes) without patients	0.70 (−0.29–1.68)	0.094	0.11	3/11
COP/Sway EC	−0.02 (−0.96–0.93)	0.840	0	2/7
YBT/SEBT/FRT	0.53 (−1.82–2.88)	0.440	1.43	3/11
YBT/SEBT without FRT	0.61 (−8.8–10)	0.560	2.2	2/10
Chronic effects vs. active controls				
COP/Sway EC	−0.01 (−4.02–4.00)	1	0.12	2/9
COP/Sway EO	−0.18 (−0.76–0.40)	0.397	0.11	4/12
COP/Sway EO in patients	−0.32 (−0.91–0.28)	0.148	0.05	3/9
COP/Sway EO static stretch	−0.03 (−1.52–1.46)	0.940	0.3	3/6
Acute effects vs. passive controls				
COP/Sway EO (without stability index outcomes)	0.20 (−0.02–0.41)	0.066	0.25	11/60
COP/Sway EO (without stability index outcomes) static stretch	0.04 (−0.12–0.21)	0.537	0	8/40
COP/Sway EO (without stability index outcomes) dynamic stretch	0.52 (−1.43–2.46)	0.183	0	2/10
COP/Sway EO (without stability index outcomes) PNF	0.31 (0.01–0.61)	0.040	0	4/11
COP/Sway EO (with stability index outcomes)	0.21 (0.02–0.39)	0.032	0.28	14/71
COP/Sway EO (with stability index outcomes) static stretch	0.06 (−0.08–0.21)	0.372	0	10/43
COP/Sway EO (with stability index outcomes) dynamic stretch	0.46 (−0.16–1.07)	0.086	0	3/11
COP/Sway EO (with stability index outcomes) PNF	0.29 (0.11–0.46)	0.009	0	6/18
COP/Sway EC	0.19 (0.08–0.31)	0.010	0	4/29
COP/Sway EC static stretch	0.21 (−0.09–0.50)	0.070	0	2/15
COP/Sway EC PNF	0.08 (−1.59–1.76)	0.635	0	2/8
YBT/SEBT	−0.04 (−2.08–2.00)	0.840	0	2/21
YBT/SEBT static stretch	−0.04 (−2.08–2.00)	0.840	0	2/21
Acute effects vs. active controls				
YBT/SEBT	0.05 (−0.27–0.37)	0.550	0	3/26

ES, effect size; 95% CI, 95% confidence interval; N, number; COP, center of pressure; EO, eyes open; EC, eyes closed; PNF, proprioceptive neuromuscular facilitation; YBT, Y-balance test; SEBT, Star Excursion balance test; FRT, Forward reach test.

Two of the studies also investigated eyes-closed conditions that both (60, 70) found no significant change. As a consequence, the quantitative analysis reveals no changes (ES: -0.02 95%CI -0.96–0.93, *p* = 0.840, τ^2^ = 0).

The three studies investigating YBT/SEBT/FRT found an increased performance in one (62) and no change in two (51, 78). However, one (78) of these two studies used the FRT, also being the only one throughout this analysis. While the meta-analytic procedure for YBT/SEBT/FRT as well as sensitivity analysis excluding the FRT (sensitivity analysis because this test differs from YBT/SEBT as it involves mostly movement in the upper body while trying to maintain balance bipedally (85)) showed moderate improvements (ES: 0.53–0.61), these effect sizes were not significant (*p* = 0.440–0.560) additionally exhibiting high heterogeneity (τ^2^ = 1.43–2.2).

As for passive control comparisons, almost all (4 out of 5) of the included active-control studies (64, 70, 76, 77) tested young or middle-aged adults (18–65 yrs. of age). The remaining study (72) investigated effects in individuals aged 65 yrs. and older. Additionally, the majority of studies (4 out of 5) took place in clinical settings (64, 70, 72, 77). However, the sway/COP eyes open patient subgroup analysis (across different diseases) resulted in non-significant effects (*p* = 0.148).

Out of the five studies with active comparison groups, four investigated static standing sway/COP parameters with eyes open for which one study (77) found an improved performance and three (64, 70, 72) no change. Two of these studies additionally determined sway/COP with eyes closed whereby one found enhanced performance (77) and one no change (70). One subgroup also investigated the effects on sway/COP with eyes open of interventions solely employing static stretching (64, 72, 77). However, all calculations exhibited non-significant effects (*p* = 0.397–1). The remaining, fifth study (76) determined YBT/SEBT outcomes and showed performance increases.

### 3.3 Acute stretching effects

Fifteen (15) studies investigated the acute effects of stretching compared to passive controls, whereby one of these (68) also incorporated active controls. For the passive control comparisons, all 15 studies incorporated healthy, young adults (18–65 yrs. of age), thus no results can be presented for older adult and patient populations.

Fourteen (14) studies investigated static standing sway/COP parameters with eyes open of which six (53, 55, 63, 65, 67, 75) found enhanced post-intervention balance performance, four (57, 58, 71, 74) no change and four (52, 59, 61, 73) a decreased performance. Pooling led to significant, small magnitude effects indicating beneficial effects for acute stretching on balance improvement (ES: 0.21 95%CI 0.02–0.39, *p* = 0.032, τ^2^ = 0.28) (see Table 2). Sensitivity analysis for stability index outcomes (excluding stability index outcomes to account for the possible impact of an index ceiling effect (81–84)) does not impact the effect size (ES: 0.20) but the level of significance (*p* = 0.066). While static and dynamic stretch subgroups yield trivial to moderate (ES: 0.04–0.52) magnitude effects that are, however, all non-significant (*p* = 0.086–0.537), both PNF subgroups for open-eyes outcomes show significant (*p* = 0.009–0.04) small magnitude effects (ES: 0.29–0.31).

Additionally, four of these 14 studies included static standing sway/COP tests with eyes closed, whereby two (53, 55) found significant positive effects, one (74) no difference and one (52) significant negative effects. The meta-analytical calculation exhibited a significant, trivial magnitude effect (ES: 0.19 95%CI 0.08–0.31, *p* = 0.010, τ^2^ = 0) when including all stretching types, with the subgroups for static and PNF stretching not reaching the level of significance (*p* = 0.070–0.635).

Moreover, two studies investigated YBT/SEBT outcomes both originating from static stretching interventions. While one found an increased performance (55), the other found no effects (68), pooling resulted in no significant change (*p* = 0.840).

All four studies (50, 54, 56, 68) incorporating active control conditions investigated young adults (18–65 yrs. of age). Out of the four studies, only one (56) used static standing sway/COP outcomes and found no effects. The remaining three studies investigated YBT/SEBT outcomes, whereby two (50, 54) showed performance increases and one (68) no change for which the meta-analytic calculation did not reach the level of significance (*p* = 0.550).

Modified funnel plot inspection indicated no publication bias for chronic effects, while some outliers caused a right shift of values. With *p* = 0.08 for chronic and *p* = 0.02 for acute effects, the Egger’s regression test supported these results.

### 3.4 Certainty about the evidence

Applying the GRADE criteria, certainty of evidence for the comparison of stretching studies on balance performance was initially rated as high due to the inclusion of (randomized) controlled trials. The level of evidence for chronic studies was downgraded for risk of bias (1 level, PEDro score of 5.4 being fair) and imprecision (1 level, few events and 95%CIs overlap no effect), resulting in low level of evidence meaning further research is very likely to impact the estimate of effect. The level of evidence for acute studies was downgraded for risk of bias (2 levels, PEDro score of 3.5 being poor), imprecision (1 level, few events and 95%CIs overlap no effect) and publication bias (1 level, as per visual inspection of funnel plots and the result of the Egger’s regression test), resulting in very low level of evidence meaning the effect estimate is very uncertain.

## 4 Discussion

This is the first systematic review with meta-analysis that found, in accordance with the GRADE score, low level evidence for the effectiveness of chronic muscle stretching for improvements in standing postural sway (significant, moderate magnitude effects). The chronic stretching effects on dynamic balance control (YBT/SEBT) were not significant. Overall, only one of the three studies (78) on chronic effects included older individuals (over age 65 years) and two (51, 70) patients with multiple sclerosis or post ankle sprain.

Acute effects of stretching interventions exhibit very low level of evidence with significant but trivial (for closed-eyes condition) to small (for open-eyes condition) effects for standing postural sway. Acute effects on dynamic balance (YBT/SEBT) following stretching interventions were non-significant. Similar to the chronic effects, only one study (71) investigated acute effects in individuals aged 65 yrs. and older.

This review also calculated the effects of studies comparing stretching to alternative, active interventions such as self-mobilization (50), jumps (56), cycling (70), trunk stabilization exercises (77) or balance training (70). Since the meta-analysis showed non-significant effects for both acute and chronic studies opposing alternative interventions, it must be questioned whether the effects found when comparing stretching to passive controls can actually be attributed to the stretching interventions or just enhanced physical activity.

The literature provides several mechanisms that could explain stretch-induced chronic and acute adaptations in balance control, including flexibility, stiffness, strength and muscle size.

### 4.1 Mechanisms of chronic effects

#### 4.1.1 Flexibility

While stretching is known as the most common flexibility training method (32) it remains uncertain if changes in flexibility actually explain the improvements in balance control. Literature speculated about the role of range of motion on postural control and balance performance as flexibility-impairments might be associated with difficulties to regain standing stability following perturbations (86). Hereby, limited ankle dorsiflexion range of motion could lead to increased subtalar joint pronation (17) that in turn could increase sway due to problems in stabilization during ankle pronation or supination. However, there might be a ceiling effect once a certain threshold of “adequate” flexibility is met, depending on how balance is measured (84). Naturally, test-specifications play a major role which is true for how balance is evaluated as well: While YBT/SEBT require a subject to lower the body’s center of gravity and keep balance standing on one foot while reaching into different directions with the other, high levels of active movement and significant changes in the body’s center of gravity are not relevant for sway/COP measurements. Therefore, it seems that YBT/SEBT performance might benefit from increased flexibility to a higher degree compared to sway/COP performance. Another factor that appears un- or underinvestigated revolves around body dimensions such as the relation of upper to lower body or thigh to lower leg in sway measurement.

The three chronic stretching studies comparing stretching with passive control conditions that found unidirectional, positive effects for all measured outcomes in the stretching groups evaluated either SEBT (51), YBT (62) or postural stability measured on a force plate during jump landing (66), which can be, depending on the definition, described as dynamic balance test conditions. Due to the small samples and effect sizes, no meaningful subgroup analyses were performed in our meta-analysis to differentiate between dynamic and static balance performance.

#### 4.1.2 Stiffness

Another parameter that could moderate the effects of stretching on balance is muscle stiffness. Stretching seems sufficient to reduce muscle stiffness parameters (87). Kim et al. (15) showed a relation of stiffness with balance performance assessed via FRT and standing balance in tandem, semi-tandem and side-by-side standing. This is in accordance with Epro et al. (16), who associated muscle-and/or tendon compliance with reduced postural control.

Unfortunately, muscle stiffness was only investigated in two of the included chronic stretching studies with passive controls showing conflicting results. While Sakai et al. (66) found decreased muscle stiffness following the intervention and increased balance performance, Gajdosik et al. (78) did neither find significant stiffness nor balance performance changes. Moreover, to the best of the authors’ knowledge, no review article investigated how stiffness affects balance.

#### 4.1.3 Maximal strength and hypertrophy

Maximal strength is associated with balance in, e.g., children and old adults (2), elite soccer players (88) and adolescent gymnasts (89). While Mühlbauer et al. (2) focused on healthy participants showing a positive relationship, no current systematic review investigated the role of maximum strength on balance in patients. However, there are several articles investigating the strength-balance relationship in patients with several indications. For instance, Hu et al. (90) showed that maximal strength positively influences balance after reconstruction of the anterior cruciate ligament while Yahia et al. (91) and Brech et al. (92) confirmed benefits on postural control in patients with multiple sclerosis and osteoporosis. This relationship was especially strong for the plantar flexors. Multiple studies (93, 94) emphasized the possibility of increasing calf muscle maximal strength and size via stretching. Muscle strength abilities might, therefore, explain the results of Kim et al. (15) and Epro et al. (16) who positively associated plantar flexor muscle strength and thickness with balance. While systematic reviews highlight the possibility of increasing maximal strength and muscle size via stretching (23, 25), only two studies from the present review measured maximal strength alongside the balance adaptations. However, both studies (51, 66) did not find significantly increased balance performance following four weeks of stretching. The maximal strength, hereby, did also not change. Thus, the influence of stretch-induced maximal strength or muscle size increases on postural control remains speculative.

However, due to its impact on general joint stability, it seems reasonable to hypothesize that an improved maximal strength capacity would help to positively influence postural sway and balance performance.

#### 4.1.4 Neuromuscular activity and proprioception

Even though results from Kubo et al. (95) reported no changes in electromyographic activity following stretching training, findings of Miyahara et al. (96) suggested potential neuromuscular adaptations. Accordingly, Nelson et al. (93) described maximal strength increases of 11% in the non-stretched, contralateral leg after a 12-week intervention period, which might be attributed to remote central nervous learning responsible for optimized muscle activity in general (97, 98). However, changes in muscle activation patterns increasing maximal strength do not automatically suggest transferability on balance performance to decrease postural sway.

Previous studies showed the involvement of reflex mechanisms, including the stretch-and H-reflex, to maintain or restore standing postural control (99). Furthermore, since the Golgi-tendon unit (100) and muscle spindle activity (101) are involved in muscle tension and muscle length control one may also expect changes in proprioception due to stretching. This may further adjust the center of pressure regulation during standing (102) and consequently, balance performance in general. This agrees with findings of Gruber et al. (103, 104) who reported reduced peak-to-peak amplitudes in soleus electromyography (EMG) activity in specific balance tasks and changes in H-reflex activity after balance training.

### 4.2 Acute effects

Due to findings showing diminished strength performance as an acute response to (static) stretch performance (43), it was hypothesized that stretching would also acutely reduce balance performance. Surprisingly, the present analysis found small but significant positive effects of acute stretching on balance performance compared to passive controls. While stretching is frequently reported to negatively influence reflex-responses acutely (105, 106), Behm et al. (33) reported these effects to dissipate within seconds after the induced stimulus. Consequently, their influence on subsequent balance performance was classified unlikely.

Since no significant differences in acute balance performance could be obtained when comparing stretching or other interventions, it must be questioned whether the effects found in comparison to passive controls are stretch-specific. This is in line with most recent evidence from a systematic review with meta-analysis (36) that did not find stretch-specific acute changes in flexibility and stiffness when compared to any other warm-up intervention and hypothesized that, at least with the current state of literature, any activity that increases core and muscle temperatures is sufficient to elicit these changes.

Muscle stiffness, when measured in a relaxed state, and flexibility, when measured passively, are both outcomes that are not directly linked to neural control. In contrast, acute changes in balance performance might comprise further mechanisms that alter, e.g., proprioception and cognitive awareness, which, however, are also known to be enhanced following any kind of warm-up activity (107, 108).

Thus, at the current state, the explanations of stretch-specific effects for acutely improved balance performance remain speculative. To specifically attribute potential stretching effects on balance, further research controlling alternative explanatory approaches is needed. Hereby, the post-test timing needs to be considered critically, as muscular fatigue significantly decreases balance performance (109).

### 4.3 Limitations

Although small effects were shown for chronic stretching on balance, there are several limitations that limit the findings’ generalizability. First, the number of studies is too small for sensible subgroup analyses of chronic and acute effects in different populations. As consequence, (a) some subgroup analyses do not reach the level of significance despite exhibiting moderate effect sizes or (b) pooled effects did not distinguish between healthy adults, patients and older adults or different stretching types/routines. The authors accounted for this by performing sensitivity analyses (excluding patient populations) or further subgroups (patients only) if possible. Though, the small number of outcomes resulted in no significant effects.

Also, high levels of heterogeneity can be observed for the included studies leading to difficulties regarding the interpretation of results. This might also be due to the heterogeneous study designs regarding the stretching interventions (intensity being oftentimes not even reported, frequency and session/overall stretching volume).

While a general warm-up prior to the stretching interventions was allowed in studies examining chronic effects, the control groups oftentimes did not receive structured interventions and thus remained non-active. Thus, it cannot be ruled out that small effects could actually be attributed to the warm-up program instead of stretching. This issue is exacerbated in one study (60) with a 15 min jogging-and jumping warm up. This study design only allows a biased interpretation of the influence of stretching.

Since no studies could be found that investigated underlying mechanisms, explanations regarding adaptations in sensorimotor or neuromuscular control mechanism can only be speculated upon. Further, the number of studies and effects in older adults settings were too small for meaningful effect pooling. Therefore, the present review calculated the effects based on heterogeneous study designs, outcomes and age groups leading to concerns regarding the validity of findings for specific populations.

The scarce number of (high quality) studies for the different age groups, stretching types and outcome measures in the field underlines the need for further investigations. Future randomized controlled stretching trials should be of high(er) methodological quality and include the investigation of different balance outcomes and a broad(er) range of potential underlying factors, such as muscle size and strength, flexibility, stiffness, neuromuscular activity and proprioception, to clarify the impact of stretching on specific outcomes such as fall prevention and motor function, especially in older adults and patients.

### 4.4 Outlook

Although some subgroups showed small to moderate magnitude balance improvements, our analysis showed no significant effects. While one possible explanation for this lack of significance is that stretching *per se* does not provide a sufficient stimulus to enhance balance, it could be argued that the load control parameters chosen in the included studies were inadequate to affect balance. Assuming structural parameters such as muscle strength/size (2, 15, 16, 88, 89) or stiffness (15, 16) as potential moderators for balance, recent reviews determined the used stretching intensity (24), weekly volume (duration per bout times frequency) (25) or supervision (110) to impact the stretch-induced effects. Therefore, the lack of significance could also be the result of low stretching intensities [e.g., stretching until point/sense of discomfort (62, 67, 75)], pain-free stretching (69), stretching until slight level of discomfort (71)), insufficient weekly volume [e.g., (78)] or frequency [e.g., (72, 77)], insufficient intervention period [e.g., (64, 76)] or performing stretching unsupervised [most did not state supervision, e.g., (69, 71, 73, 76, 78)], which could, in turn, be associated with insufficient intensity (110). In contrast, since Konrad et al. (32) did not find these parameters to affect stretching results on flexibility, it could be speculated that flexibility might not be the primary outcome to affect balance. As a consequence, future studies should adopt research designs that have the potential to actually affect strength and hypertrophy (stretching durations >15 min per bout on more than 5 days per week for >6 weeks (25) or moderate stiffness (thus, be performed supervised (110)) to investigate the potential role of stretch-mediated effects in balance improvements to provide robust results.

Assuming muscle strength, size and flexibility to moderate balance, there are more common training interventions to target, for example maximal strength. Indeed, LaCroix et al. (20) found resistance training to be beneficial for balance capabilities, especially when supervised, while Hu et al. (111) showed that plantar massage as well as whole body vibration training also improved static balance with comparable effect sizes (0.54 and 0.66, respectively). Obviously, the highest effect sizes were reported for specific balance training, with, for example, ES = 0.83 for dynamic balance (112). It must be noted that there are several concurrent training approaches with partly very different effect sizes in specific patient groups such as chronic ankle instability patients (113, 114), Parkinson disease (115, 116) or back pain patients (117). Therefore, it seems necessary to investigate specific balance interventions for specific group settings and explore the outcomes to determine the most effective way to enhance balance in each of these. However, due to the currently limited number of stretching studies, direct subgroup comparisons will not reveal any valuable insights.

## 5 Conclusion

Due to the limited number of studies, high methodological heterogeneity as well as (very) low levels of certainty in the found evidence according to the GRADE score, it is nearly impossible to provide conclusive statements for the practical relevance of stretching for balance control. Reduced muscle strength in the lower leg is associated with ankle instability, however, especially immobilized and conditioned populations face obstacles when aiming to implement resistance training in an effective and safe manner. Especially in populations with limitations in motor function, stretching could provide a safe training intervention which could be implemented as an unsupervised training. However, the practical applicability of a resistance training substitution through stretching must be further investigated using higher stretching volumes in future randomized controlled trials that are of high(er) methodological quality. Consequently, stretching performed with the required volume and intensity might not only be relevant in counteracting immobilization-related muscle strength and size decreases (118), but could help prevent functional performance impairments, i.e., balance.

## Data availability statement

The original contributions presented in the study are included in the article/Supplementary material, further inquiries can be directed to the corresponding author.

## Author contributions

LL: Conceptualization, Data curation, Formal analysis, Methodology, Supervision, Writing – original draft, Writing – review & editing. AZ: Conceptualization, Methodology, Writing – review & editing, Supervision. GP: Data curation, Writing – review & editing, Formal analysis. MO: Data curation, Writing – review & editing, Formal analysis. DJ: Data curation, Software, Visualization, Writing – review & editing. KW: Data curation, Formal analysis, Software, Supervision, Visualization, Writing – original draft, Writing – review & editing, Methodology.
