# Supplementary material for: Acute and chronic effects of stretching on balance: a systematic review with multilevel meta-analysis
Source: Front Med (Lausanne). 2024 Sep 13;11:1451180. doi: 10.3389/fmed.2024.1451180 (PMC11427387; doi:10.3389/fmed.2024.1451180)
Supplement: Supplementary file 1 [file Table_1.docx]

**Supplemental material**

**Search strings**

Web of Science

ALL=((stretch*) AND (balance OR "postural control" OR stability OR proprioception))

Filter: Web of Science Categories: Sports Sciences or Physiology

Languages: German or English

Hits: 775

Scopus

TITLE-ABS-KEY(stretch*) AND TITLE-ABS-KEY(balance OR "postural control" OR stability OR proprioception)

TITLE-ABS-KEY ( stretch* ) AND TITLE-ABS-KEY ( balance OR "postural control" OR stability OR proprioception ) AND ( LIMIT-TO ( SUBJAREA , "HEAL" ) OR LIMIT-TO ( SUBJAREA , "MEDI" ) ) AND ( LIMIT-TO ( LANGUAGE , "English" ) OR LIMIT-TO ( LANGUAGE , "German" ) ) AND ( LIMIT-TO ( EXACTKEYWORD , "Human" ) OR LIMIT-TO ( EXACTKEYWORD , "Humans" ) )

(Limited to Medicine & Health Professions

Limited to Human & Humans

Limited to English & German)

**PEDro scale criteria** (last amended 21 June 1999)

1. eligibility criteria were specified (not used to calculate the PEDro score)
2. subjects were randomly allocated to groups (in a crossover study, subjects were randomly allocated an order in which treatments were received)
3. allocation was concealed
4. the groups were similar at baseline regarding the most important prognostic indicators
5. there was blinding of all subjects
6. there was blinding of all therapists who administered the therapy
7. there was blinding of all assessors who measured at least one key outcome
8. measures of at least one key outcome were obtained from more than 85% of the subjects initially allocated to groups
9. all subjects for whom outcome measures were available received the treatment or control condition as allocated or, where this was not the case, data for at least one key outcome was analysed by “intention to treat”
10. the results of between-group statistical comparisons are reported for at least one key outcome
11. the study provides both point measures and measures of variability for at least one key outcome

**Tab. A: PEDro score**

| **Study** | **2.** | **3.** | **4.** | **5.** | **6.** | **7.** | **8.** | **9.** | **10.** | **11.** | **Score** |
| --- | --- | --- | --- | --- | --- | --- | --- | --- | --- | --- | --- |
| Akdag et al. (50) | Y | N | Y | N | N | N | N | N | Y | Y | 4 |
| Alahmari et al. (51) | Y | Y | Y | N | N | Y | Y | Y | Y | Y | 8 |
| Alimoradi et al. (52) | Y | N | Y | N | N | N | N | N | Y | Y | 4 |
| Ayân et al. (53) | Y | Y | N | N | N | N | N | N | Y | Y | 4 |
| Behm et al. (54) | Y | N | Y | N | N | N | N | N | Y | Y | 4 |
| Coratella et al. (55) | Y | N | Y | N | N | Y | N | N | Y | Y | 5 |
| Costa et al. (56) | Y | N | N | N | N | N | N | N | Y | Y | 3 |
| Espí-López et al. (57) | Y | Y | Y | N | N | Y | Y | Y | Y | Y | 8 |
| Fontana Carvalho et al. (58) | Y | Y | Y | N | N | N | N | Y | N | Y | 5 |
| Gajdosik et al. (59) | Y | Y | Y | N | N | N | Y | N | Y | Y | 6 |
| Ghram et al. (60) | Y | N | N | N | N | N | Y | N | Y | Y | 4 |
| Grahm et al. (61) | Y | N | N | N | N | N | N | N | Y | Y | 3 |
| Jouira et al. (62) | N | N | Y | N | N | N | N | N | Y | Y | 3 |
| Jung et al. (63) | Y | N | N | N | N | Y | Y | Y | Y | Y | 6 |
| Kim et al. (64) | N | N | N | N | N | N | N | N | Y | Y | 2 |
| Leblebici et al. (65) | Y | N | N | N | N | N | N | N | N | Y | 2 |
| Lim et al. (66) | Y | Y | N | N | N | N | N | N | Y | Y | 4 |
| Lima et al. (67) | N | N | N | N | N | N | N | N | N | Y | 1 |
| Mel’nikov et al. (68) | N | N | N | N | N | N | N | N | Y | Y | 2 |
| Oba et al. (69) | Y | N | Y | N | N | N | N | N | Y | Y | 4 |
| Oba et al. (70) | Y | N | Y | N | N | N | N | N | Y | Y | 4 |
| Park et al. (71) | Y | N | Y | N | N | Y | Y | Y | Y | Y | 7 |
| Ryan et al. (72) | Y | N | N | N | N | N | N | N | Y | Y | 3 |
| Sakai et al. (73) | Y | N | Y | N | N | N | Y | N | Y | Y | 5 |
| Szafraniec et al. (74) | N | N | N | N | N | N | N | N | Y | Y | 2 |
| Thomas et al. (75) | Y | N | Y | N | N | Y | Y | N | Y | Y | 6 |
| Todde et al. (76) | N | N | Y | N | N | N | N | N | Y | Y | 3 |
| Tollar e al. (77) | Y | Y | Y | N | N | Y | Y | N | Y | Y | 7 |
| Wallmann et al. (78) | N | N | N | N | N | N | Y | N | Y | Y | 3 |

**Legend.** N = No, Y = Yes
